# Pathogenic potential of interferon αβ in acute influenza infection

**DOI:** 10.1038/ncomms4864

**Published:** 2014-05-21

**Authors:** Sophia Davidson, Stefania Crotta, Teresa M McCabe, Andreas Wack

**Affiliations:** 1 Division of Immunoregulation, MRC National Institute for Medical Research, Mill Hill, London NW7 1AA, UK

## Abstract

Influenza symptoms vary from mild disease to death; however, determinants of severity are unclear. Type I interferons (IFNαβ) are recognized as key antiviral cytokines. Here we show that, surprisingly, influenza-infected 129 mice have increased lung damage, morbidity and mortality, yet higher levels of IFNαβ, than C57BL/6 mice. Consistently, IFNα treatment of influenza-infected C57BL/6 mice increases morbidity. IFNαβ receptor deficiency in 129 mice decreases morbidity, lung damage, proinflammatory cytokines and lung-infiltrating inflammatory cells, and reduces expression of the death-inducing receptor DR5 on lung epithelia and its ligand TRAIL on inflammatory monocytes. Depletion of PDCA-1+ cells or interruption of TRAIL-DR5 interaction protects infected 129 mice. Selective lack of IFNαβ signalling in stromal cells abolishes epithelial DR5 upregulation and apoptosis, reducing host susceptibility. Hence, excessive IFNαβ signalling in response to acute influenza infection can result in uncontrolled inflammation and TRAIL-DR5-mediated epithelial cell death, which may explain morbidity and has important implications for treatment of severe disease.

## Abstract (web summary)

Interferon αβ(IFNαβ) is known as a potent anti-viral factor, yet its role in influenza infection remains controversial. Here, the authors show that the IFNαβ response is a critical host factor, which, when excessive, causes strong inflammation and severe disease in a mouse model of acute influenza infection.

## Graphical abstract

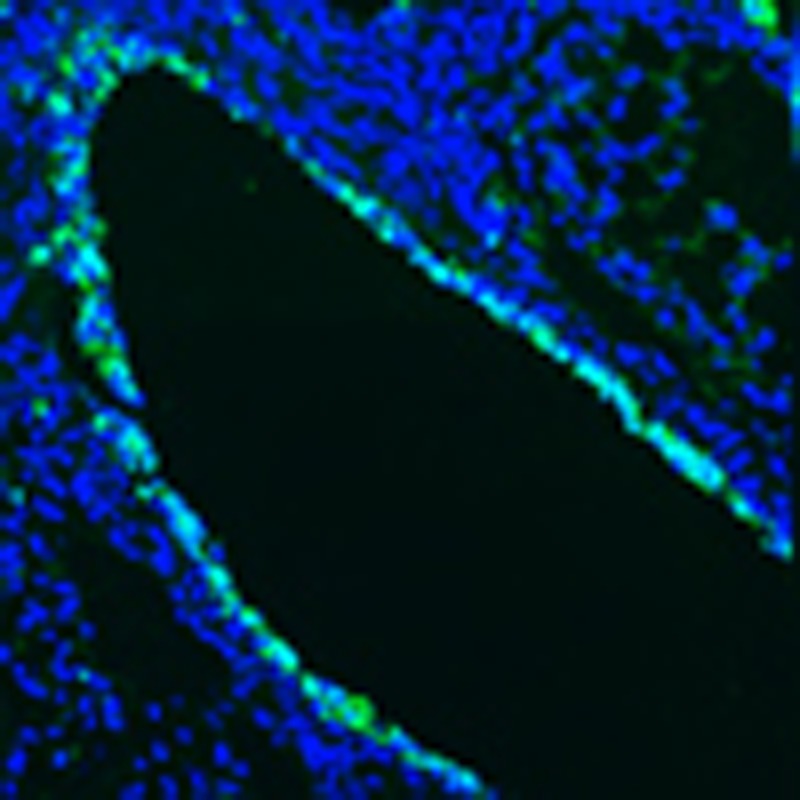

Variations in influenza-induced disease severity in humans are usually attributed to the virulence of different influenza virus strains or the age or immune status of infected individuals. The role of genetically determined host factors is less well understood^1^,^2^, and only recently has an influenza virus restriction factor, IFITM3, been identified in human populations^3^. Familial clusters of severe influenza suggest host-dependent susceptibility in humans^4^,^5^,^6^, and studies in outbred and inbred mice show a wide range of host-specific genetic susceptibility with many candidate genes proposed to contribute to susceptibility or resistance^4^,^7^.

Type I interferon or IFNαβ is widely recognized to have antiviral function *in vitro*^8^,^9^,^10^, and lack of IFNαβ signalling in receptor (IFNαβR)-deficient mice increases susceptibility to many viruses other than influenza virus^11^. However, the role of IFNαβ in restricting influenza infection *in vivo* is less clear^12^,^13^,^14^,^15^,^16^,^17^. In particular, while some studies suggest a protective role of IFNαβ, others found no effect. Possible reasons for the discrepancies between these studies are infection with different virus doses and strains, including those with unusual tissue tropism such as WSN, and usage of either IFNαβR−/− or signal transducer and activator of transcription1−/− (STAT1−/−) mice as models of IFN signalling deficiency on C57BL/6, 129Sv/Ev, CD1 or mixed mouse backgrounds, making comparisons within and between studies difficult. We now know that STAT1 is a transcription factor that acts alone or together with other STAT family members downstream of the receptors for IFNαβ, -γ and -λ and a number of other cytokines that have wide-ranging effects, such as interleukin (IL)-6, IL-10 and IL-27 (ref. 18). The high susceptibility of STAT1−/− mice^13^,^14^, therefore, may be the result of their inability to respond to a range of cytokines. In particular IFNλ, or type III IFN, is a more recently discovered antiviral IFN that triggers STAT1 activation through engagement of an independent receptor found primarily on epithelial cells of both the respiratory and gastrointestinal tract. In studies where IFNαβ and IFNλ receptor-deficient mice were infected with influenza^12^, only the absence of both receptors caused marked loss of virus control.

The specific role of IFNαβ in influenza infection is still unclear. Type I IFNs are known to have potent immunomodulatory effects, which may interfere with or supersede their antiviral function. For instance, it was recently shown that IFNαβ can inhibit inflammasome activation^19^, a pathway that was suggested to have an important role in the immune response to influenza^20^. The immunomodulatory properties of IFNαβ can also have a negative impact on the outcome of bacterial infection. Thus, IFNαβR-deficient mice were more resistant to infection by Listeria and Trypanosoma^21^,^22^, and increased IFNαβ levels at least partly explain the increased severity of bacterial infection post influenza infection or poly I:C treatment^23^,^24^. To summarize, under what conditions IFNαβ is protective against influenza remains an open question, and the relative contributions of its antiviral and immunomodulatory properties to the overall outcome of influenza-induced disease are yet to be determined.

Tumour necrosis factor (TNF)-related apoptosis-inducing ligand (TRAIL), also known as Apo2L, is a member of the TNF superfamily (TNFSF10) of cytokines and initially gained attention as an attractive anticancer drug candidate for its ability to induce apoptosis specifically in transformed cells but not in normal, untransformed tissues^25^. Five TRAIL receptors have been described in both humans and mice. However, it is only TRAIL-R1 and TRAIL-R2 (murine DR4 and DR5, respectively) in humans and only DR5 in mice that contain death domains and therefore induce cell death on TRAIL engagement, while the remaining TRAIL receptors are considered decoys^26^. Recently, it was shown that immune cells and other nontransformed cells can upregulate both TRAIL and DR5, mostly in situations of infection and inflammation^27^, and, importantly, IFNαβ-induced TRAIL expression was linked to T-cell death in chronic HIV infection^28^.

Here we demonstrate across a number of mouse strains a direct correlation between high morbidity and mortality and high IFNαβ levels in response to influenza infection. We show directly that, in susceptible 129 mice, lack of the IFNαβ receptor reduces morbidity and mortality, clearly indicating that excessive IFNαβ contributes to disease. Furthermore, we find that 129 mice have increased numbers of hyper-responsive pDCs and that influenza-induced IFNαβ leads to high levels of inflammatory cytokines and chemoattractants, massive inflammatory cell recruitment and strong expression of TRAIL on inflammatory monocytes and DR5 on epithelia whose interaction mediate lung tissue damage. Depletion of PDCA-1+ cells or blockage of the TRAIL-DR5 interaction protects 129 mice from severe influenza. In conclusion, we delineate a host-specific pathway responsible for high immunopathology and susceptibility after influenza infection, with excessive IFNαβ derived from PDCA-1+ cells being a central player and TRAIL-DR5 interaction a downstream mediator of morbidity.

## Results

### IFNαβ and -λ levels correlate with influenza disease severity

**Figure 1:**
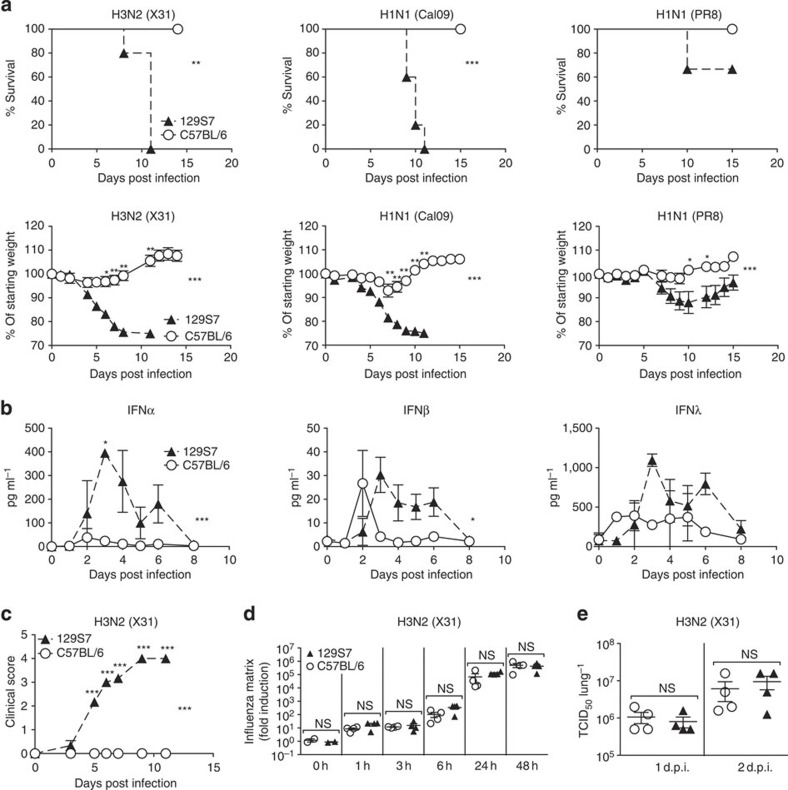
Increased influenza susceptibility of 129S7 mice correlates with higher concentrations of type I and type III interferons. (**a**) 129S7 (filled triangles) or B6 (open circles) mice were infected i.n. by indicated influenza virus strains: X31 (800 TCID_50_), Cal09 (1,000 TCID_50_) or PR8 (5 TCID_50_), and weight loss and mortality recorded. (**b**–**d**) 129S7 and B6 mice were infected with X31(800 TCID_50_). (**b**) IFN levels in BAL fluid were measured by ELISA, and (**c**) mice were scored for clinical symptoms (as described in methods). (**d**,**e**) Viral presence in infected lungs was quantitated (**d**) by qPCR for the X31 Matrix gene in RNA from whole lungs or (**e**) by virus titration. Graphs show mean±s.e.m. and are representative of 2–5 independent experiments where *n*=3 for ELISA data, *n*=4 for qPCR and virus titration and *n*≥6 for weight loss and mortality. ***P<0.0001, **P<0.001, *P<0.01 by two-way ANOVA with Bonferroni post-tests (weight loss and ELISA) where symbols on the right of graphs indicate statistical significance of the whole curve, as tested by two-way ANOVA, and those above specific days indicate significance found by post test, Log-rank (Mantel-Cox) Test (survival) or Mann–Whitney test (viral quantification).

We compared influenza-induced disease in 129/SvEvBrd-Hprt^b-m2^ (129S7 or 129) and C57BL/6 (B6) mice and observed marked differences in morbidity and mortality after infection with the low pathogenicity H3N2 influenza strain X31, the pandemic H1N1 strain Cal09 and the classical H1N1 strain PR8 (Fig. 1a). 129 mice developed pronounced clinical symptoms including piloerection, hunched posture, reduced movement and labored breathing (Fig. 1c), associated with marked weight loss and ultimately reaching clinical end point for humane euthanasia, which we found in independent experiments to correlate with host mortality. Importantly, at the virus doses used to elicit disease in 129 mice, all B6 mice survived and lost little to no weight. This difference was observed over several logs of viral inoculate of X31, PR8 and Cal09. Independent egg-derived and MDCK-derived preparations of the X31 virus gave similar results, and differences between the mouse strains were observed both for males and females.

Other 129 substrains tested included the 129 X1, S2, S5, S6 and S8 strains and comprised mice bred on site in different animal units and mice imported from commercial breeders. All 129 substrains that we tested showed a much higher susceptibility than B6 mice (Supplementary Fig. 1). Among the immunological parameters assessed, we found that influenza-induced levels of the antiviral cytokines IFNα, -β and -λ in bronchioalveolar lavage (BAL) fluid were markedly higher in susceptible 129 than in resistant B6 mice at most time points post infection for all influenza strains tested (Fig. 1b, Supplementary Fig. 2a). Similar results were obtained from lung homogenates.

Furthermore, we tested a range of independent mouse strains and found that BALB/c mice followed the disease course of resistant B6 mice and had comparably low IFN levels (Supplementary Fig. 3a,b), whereas CBA/J and DBA/2 mice exhibited high susceptibility to influenza, resembling the 129 strains, and also displayed sustained, high IFNα, -β and -λ levels throughout infection (Supplementary Fig. 3c–f). In addition, (B6 × 129)F1 mice had intermediate IFN levels and susceptibility to influenza (Supplementary Fig. 3g,h). In conclusion, IFNαβ levels correlate directly, not inversely with influenza-induced morbidity and mortality across a wide range of mouse genetic backgrounds and virus strains.

The differences in IFNαβ production after infection could be host intrinsic or a consequence of more rapid virus replication early in infection, leading to a stronger stimulus to produce IFN. To address this, we measured virus present in the lungs of mice by qRT–PCR for influenza matrix RNA from 1 to 48 h post infection (Fig. 1d) and by virus titrations on whole-lung homogenate (Fig. 1e, Supplementary Fig. 2b). 129 and B6 mice had comparable virus titres between 1 h and 3 days post infection, a period where differences in IFNα production have already been established. We therefore conclude that the host differences in IFN levels shown here are not a consequence of higher virus titres early in infection.

### Innate immunity mediates strain specific susceptibility

**Figure 2:**
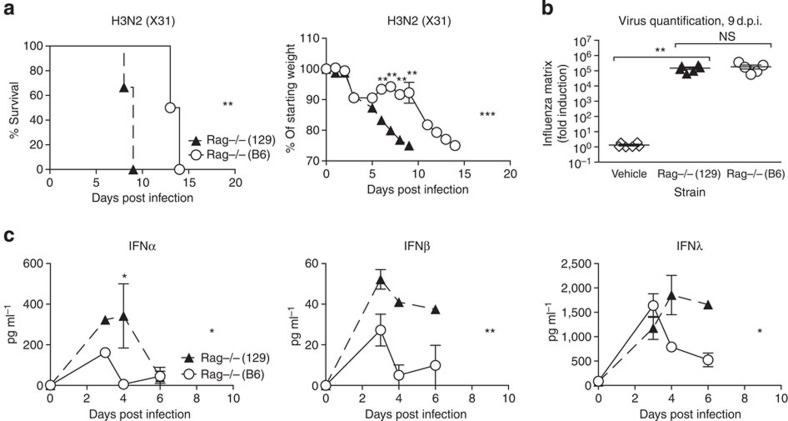
Increased susceptibility of 129 mice is independent of adaptive immunity. (**a**–**c**) Rag-deficient 129 (filled triangles) or B6 (open circles) mice were infected intranasally with 800 TCID_50_ of X31, and (**a**) weight loss and morbidity recorded. (**b**) Virus RNA present in the lung on day 9 was determined by RT–PCR on total lung, and (**c**) IFN protein was quantified by ELISA in BAL fluid. Graphs show mean±s.e.m. and are representative of two independent experiments where *n*=5–6 for weight loss and survival and *n*=3–5 for ELISA and qPCR. ***P<0.0001, **P<0.001, *P<0.01 by two-way ANOVA with Bonferroni post tests (weight loss and ELISA) or Log-rank (Mantel-Cox) Test (survival) or Mann–Whitney test (viral quantification). The symbols on the right of graphs indicate statistical significance of the whole curve, as tested by two-way ANOVA.

To understand whether the observed host differences in susceptibility are due to innate or adaptive immunity, we compared Rag-deficient 129 and B6 mice, which are deficient for both B and T cells. While all Rag−/− mice eventually succumb to influenza infection, Figure 2a demonstrates that infected Rag−/−(129) mice, like their wild-type (wt) counterparts, lost weight and reached clinical end point more rapidly than Rag−/−(B6) mice and had higher concentrations of IFNα, -β and -λ in their BAL (Fig. 2c). Virus quantification at 9 days post infection (d.p.i.), when Rag−/−(129) mice were at clinical end point yet all Rag−/−(B6) mice were still alive, showed no differences in virus amounts in the lung (Fig. 2b). We conclude that increased susceptibility to influenza in 129 mice is likely due to innate immune responses, does not correlate with virus control, and is associated with sustained high levels of the antiviral cytokines IFNα, -β and -λ.

### IFNαβ mediates influenza-induced morbidity

**Figure 3:**
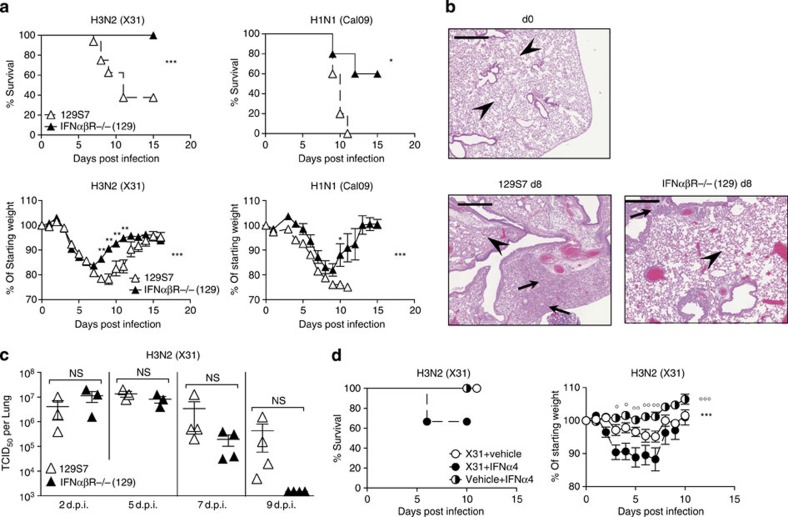
Lack of type I IFN signalling is protective in 129S7 mice. (**a**–**c**) 129 (open triangles) or IFNαβR−/− (filled triangles) mice were infected i.n. with X31, 800 TCID_50_ or Cal09, 330 TCID_50_. (**a**) Weight loss and mortality were measured. (**b**) Haematoxylin & Eosin-stained sections (scale bar 50 μm) from lungs taken at days 0 and 8 post infection. Arrows indicate leukocyte infiltrate and arrowheads indicate intact alveolar structure. (**c**) Virus titres in lung homogenates taken at the indicated time points were measured by TCID_50_ determination on MDCK cells. (**d**) B6 mice were infected i.n. with X31 (8000 TCID_50_ in 30 μl) or treated with vehicle control on d0 and subsequently treated with mammalian IFNα4 (3.5 × 10^4^IU  per 200 μl i.p.), or vehicle control every 24 h from d1 to d6. Weight loss and morbidity were recorded over time. Graphs show mean±s.e.m. and are representative of 2–4 independent experiments where *n*=5–6 for weight loss and survival and *n*=4 for viral titration. X31+IFNα4:X31+Vehicle Control *, X31+IFNα4:Vehicle Control+IFNα4 ^○^. *** or ^○○○^*P*<0.0001, ** or ^○○^*P*<0.001, * or ^○^*P*<0.01 by two-way ANOVA with Bonferroni post-tests (weight loss) or Mann–Whitney test (viral quantification) or Log-rank (Mantel–Cox) Test (survival). The symbols on the right of graphs indicate statistical significance of the whole curve, as tested by two-way ANOVA.

The increased levels of IFN in the more susceptible mouse strain led us to investigate directly the effect of IFNα and -β signalling in the 129 background. Since all IFNα and -β types exclusively signal through the IFNαβR^11^, we compared 129 mice deficient for this receptor to their respective wt controls. Genetic removal of IFNαβ signalling in 129 mice leads to increased resistance to infection by the low pathogenicity H3N2 strain X31 and the pandemic H1N1 influenza strain Cal09 (Fig. 3a). Differences in morbidity were reflected in lung tissue damage, where lungs from 129 mice at day 8 post infection show a high cell infiltrate leading to massive obstruction of alveolar airspaces. In IFNαβR−/− (129) mice, infiltrate into the lung is reduced and restricted to peribronchial and perivascular regions, and the alveolar structure is better preserved compared with the parental strain (Fig. 3b). In line with susceptibility data, there is a trend towards delayed virus clearance in 129 mice compared with IFNαβR−/−(129) mice (Fig. 3c); however, this was not statistically significant. Antibody titres at 9 d.p.i. also show small but not significant differences when measured by microneutralization assay (Supplementary Fig. 4a), confirming previously published results^15^ on the role of IFN for antibody production.

### IFNαβ is redundant for antiviral gene induction in epithelia

**Figure 4:**
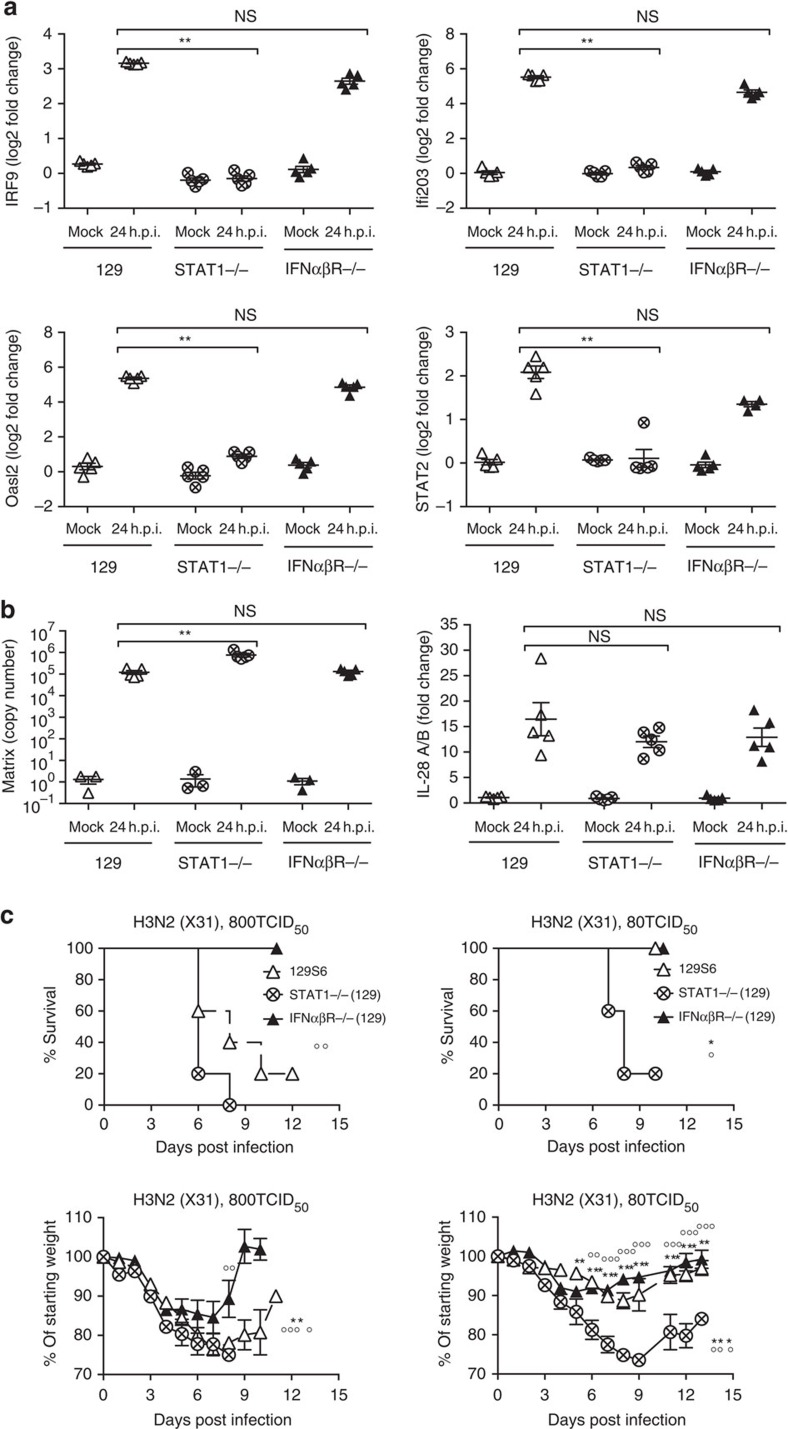
STAT1 is required for ISG induction and resistance on influenza infection of 129 mice. (**a**,**b**) STAT1−/−(129), IFNαβR−/−(129) and 129 epithelial cell cultures were infected with X31 at a MOI of 1. At 24 h post infection upregulation of (**a**) Oasl2, STAT2, IRF9, Ifi203, (**b**) IL-28 (IFNλ) mRNA and replication of X31 was assessed by RT–PCR. (**c**) STAT1−/−(129) (crossed circles), IFNαβR−/−(129) (filled triangles) and 129 mice (open triangles) were infected i.n. with 800 TCID_50_ (upper panels) or 80 TCID_50_ (lower panels) of X31. Weight loss and survival were recorded throughout infection. Graphs show mean±s.e.m. and are representative of two independent experiments where *n*=5 for qPCR and *n*=6 for weight loss and survival. 129:STAT1−/−(129) * and IFNαβR−/−(129):STAT1−/−(129) ^○^, where **** or ^○^^○^^○^^○^*P*<0.00001, ** or ^○^^○^*P*<0.001, *P<0.01 by 2-way ANOVA (weight loss), Log-rank (Mantel-Cox) Test (survival) or Mann–Whitney test (RT–PCR quantification). The symbols on the right of graphs indicate statistical significance of the whole curve, as tested by two-way ANOVA.

Since IFNαβ is able to induce antiviral programmes, it could be argued that loss of IFNαβ signalling should result in loss of antiviral gene induction and therefore facilitate viral propagation in infected tissues. However, interferon-stimulated genes (ISGs) with antiviral function were similarly upregulated on infection with the X31 virus strain in primary airway epithelia from 129 and IFNαβR−/−(129) mice (Fig. 4a), consistent with the suggested redundancy of IFNαβ and IFNλ in epithelial cells^29^,^30^. In contrast, STAT1−/−(129) epithelia were unable to upregulate ISGs on influenza infection (Fig. 4a).

Consistent with ISG induction, by 24 h post infection, virus titres in IFNαβR−/−(129) epithelia were similar to those in wt epithelia and were significantly increased in STAT1−/− epithelia (Fig. 4b). As expected, epithelia of all genotypes showed influenza-induced upregulation of genes such as IFNλ (IL28, Fig. 4b), which are upstream of IFN-mediated STAT1 induction. Similar results were obtained using PR8 (Supplementary Fig. 5a–c).

From these data, we conclude that the antiviral programme determined by ISG induction is intact in IFNαβR−/−(129) lung epithelia, while in STAT1−/− epithelia ISGs are not induced and virus control is impaired. Therefore, the inability of STAT1−/− mice to upregulate ISGs with antiviral function may contribute to the high susceptibility of STAT1−/−(129) mice to influenza infection (Fig. 4c). In contrast, as IFNαβ signalling alone has negligible impact on viral control in epithelia, the deleterious effects of IFNαβ are likely due to its immunomodulatory function.

To understand whether interventions to increase IFNαβ levels would render resistant mice more susceptible, we infected B6 mice and treated them with exogenous IFNα4. These mice showed increased morbidity compared with infected B6 mice that did not receive IFNα treatment (Fig. 3d). Thus, removal of IFNαβ signalling during influenza infection ameliorates disease in 129 mice, and exogenous administration of IFNα to B6 mice increases disease rather than aiding the control of viral spread. Together, these data suggest that high-dose IFNαβ responses contribute to influenza pathology, while induction of antiviral ISGs is little affected in the absence of IFNαβ signalling at the site of viral replication.

### IFNαβ mediates influenza-induced inflammation

**Figure 5:**
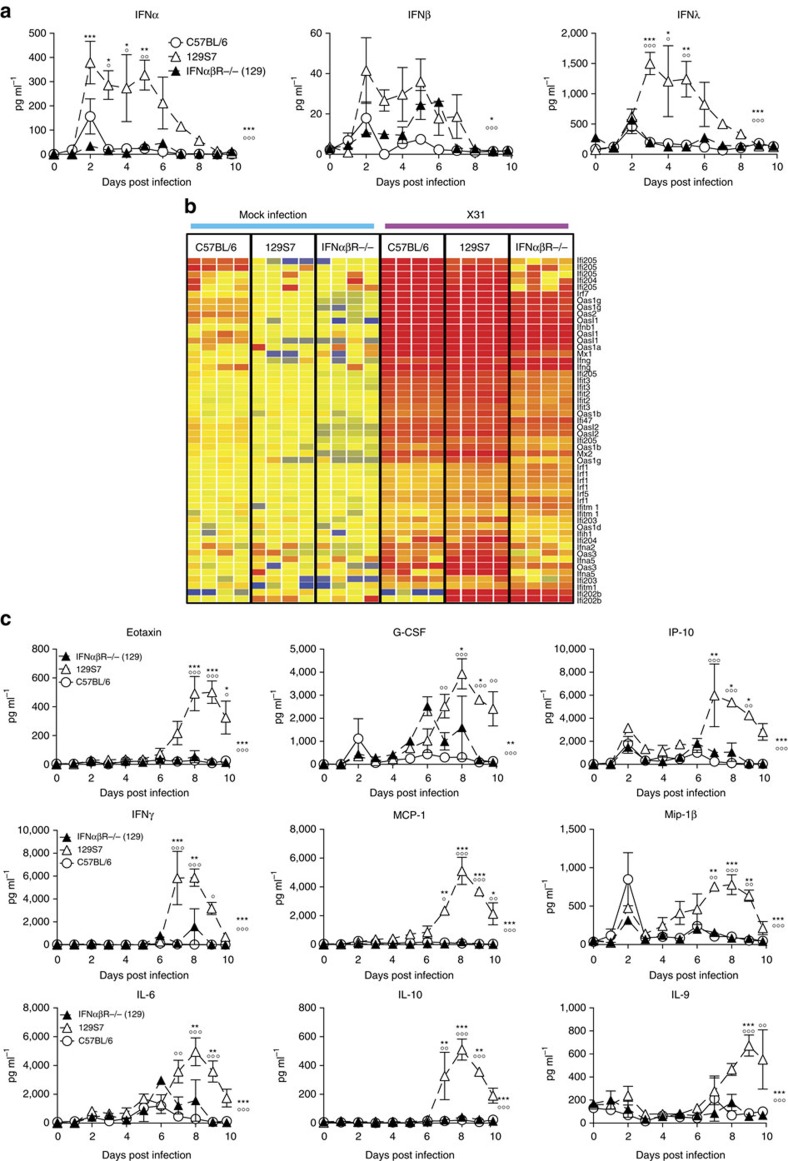
Type I IFN signalling in 129S7 mice leads to high concentrations of proinflammatory cytokines in BAL. (**a**–**c**) 129S7 (open triangles), IFNαβR−/−(129) (filled triangles) or B6 (open circles) mice were infected i.n. with 800 TCID_50_ of X31. (**a**) IFN levels in BAL fluid were measured by ELISA and (**c**) specified pro-inflammatory cytokine concentrations were quantified by Multiplex. (**b**) Heatmap displaying selected significantly regulated antiviral response genes. Total RNA from mock and X31-infected lung was analysed using Affymetrix Mouse Genome 430 2.0 microarrays at 5 days post infection. Supervised analysis was performed using statistical filtering (≥fourfold change relative to mock-infected C57BL/6; 2-way ANOVA, *P*<0.01, Benjamini-Hochberg multiple test correction). Graphs show mean±s.e.m. and are representative of two independent experiments where *n*=3–4. 129:IFNαβR−/−(129) * and 129:B6 ^○^, where *** or ^○^^○^^○^*P*<0.0001, ** or ^○^^○^*P*<0.001, *P<0.01 by two-way ANOVA with Bonferroni post tests. The symbols on the right of graphs indicate statistical significance of the whole curve, as tested by two-way ANOVA.

To better understand the immunopathological effect of high-level IFNαβ, we investigated inflammatory cytokines and immune cells in infected 129 wt and IFNαβR−/− lungs. Interestingly, levels of both IFNαβ and IFNλ in IFNαβR−/−(129) BAL were lower than in wt 129 BAL and comparable to those found in resistant wt B6 (Fig. 5a). This indicates that an IFNαβ signal is required to maintain the high levels of both IFNαβ and IFNλ that we observe in the 129 background. Nevertheless, these levels of IFN are sufficient for the induction of selected ISGs *in vivo*. As shown in Fig. 5b, ISG upregulation found in infected IFNαβR−/−(129) lungs closely reflects the ability of IFNαβR−/−(129) epithelia to upregulate ISGs in response to influenza *in vitro* (Fig. 4a and Supplementary Fig. 5a).

Apart from their antiviral functions, IFNs have potent immunomodulatory effects, which include the induction of chemokines and cytokines and the activation of a wide range of immune cells. We therefore analysed cytokine and chemokine concentrations in the BAL fluid of infected B6, 129 and IFNαβR−/−(129) mice, and found massively increased amounts of the chemoattractants MCP-1, Mip-1β, IP-10, IL-6, Eotaxin and others from 4 to 6 days onwards in 129 mice compared with B6 mice. Interestingly, IFNαβR−/−(129) mice did not show an increase in proinflammatory cytokines as seen in the parental strain but resemble more the resistant B6 mice (Fig. 5c), demonstrating that an IFNαβ signal is required for the pro-inflammatory cytokine milieu found in 129 mice. Along with the massive upregulation of pro-inflammatory cytokines, we also find elevated levels of IL-10 in 129 mice, which may contribute to disease severity by attenuating specific effector functions, even though virus control is not suppressed in 129 mice (Fig. 3c).

**Figure 6:**
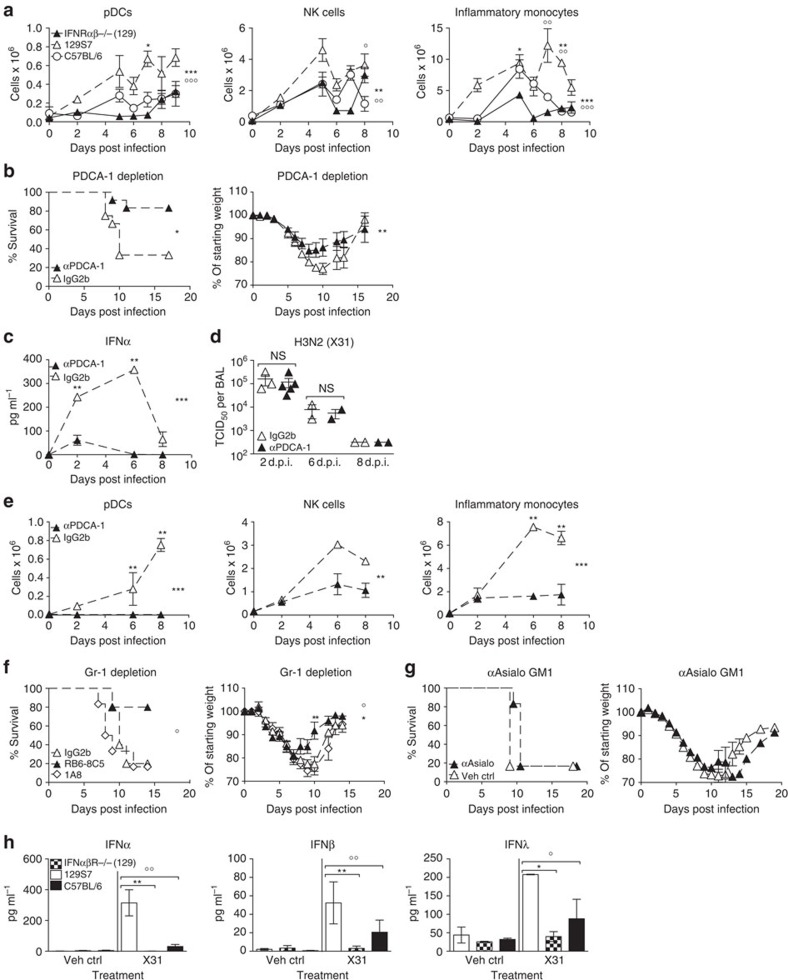
IFNαβ derived from pDCs and other PDCA-1+ cells mediates inflammation and morbidity in infected 129S7 mice. (**a**) 129S7 (open triangles), IFNαβR−/−(129) (filled triangles) or B6 (open circles) mice were infected with X31 and flow cytometric quantification of pDCs, NK cells and inflammatory monocytes in the lung was performed. 129S7:IFNαβR−/−(129) *, 129S7:B6 ^○^. (**b**–**e**) 129S7 mice were treated with depleting mAb αPDCA-1 or isotype control as indicated, (**b**) weight loss and mortality were measured, (**c**) IFNα protein in BAL fluid was quantified by ELISA, (**d**) viral titre in BAL fluid were determined and (**e**) cell recruitment was assessed as in **a**. (**f**) 129S7 mice were treated with depleting mAbs RB6-8C5 (filled triangles), 1A8 (open diamonds) or isotype control (open triangles) then infected with X31. (**g**) 129S7 mice were treated with αAsialo GM1 (filled triangles) or Vehicle Control (open triangles) then infected with X31. (**f**,**g**) Mortality and weight loss were recorded. (**h**) BM-derived pDCs from 129 (open columns), IFNαβR−/−(129) (checked columns) and B6 (filled columns) mice were stimulated *in vitro* with X31. At 24 h supernatants were collected and concentrations of IFNα, β and λ were measured by ELISA. Graphs show mean±s.e.m. and are representative of 2–3 independent experiments where *n*=2–4 for cellular recruitment, virus titration and ELISA, *n*=5–6 for weight loss and survival except for **b** where data are pooled from two experiments (*n*=12). *** or ^○^^○^^○^*P*<0.0001, ** or ^○^^○^*P*<0.001, *P<0.01 by two-way ANOVA with Bonferroni post tests (cell counts, weight loss and ELISA time course), Log-rank (Mantel-Cox) Test (survival) or Mann-Whitney test (pDC supernatants). The symbols on the right of graphs indicate statistical significance of the whole curve, as tested by two-way ANOVA.

NK cells, inflammatory monocytes and CD8 T cells are among the cellular targets of IFNs and the chemoattractants found elevated in 129 mice. FACS analysis of the cellular infiltrate into infected B6, 129 and IFNαβR−/−(129) lungs revealed that at all time points examined, IFNαβR−/−(129) mice have lower frequencies and numbers of inflammatory monocytes and NK cells than 129 mice (Fig. 6a). Furthermore, numbers of these inflammatory cells were comparable between lungs of the resistant IFNαβR−/−(129) and B6 strains. In contrast, frequencies and numbers of total and of influenza-specific CD8 T cells as measured by NP_366–374_ H-2D^b^ tetramer staining were unchanged between 129, IFNαβR−/−(129), and B6 mice (Supplementary Fig. 4b). This further illustrates that IFN-dependent innate immune responses are linked to the increased susceptibility observed in 129 mice and places IFNαβ upstream of the strong induction of pro-inflammatory cytokines and inflammatory cell recruitment.

### Abundant hyper-reactive 129 pDCs produce excessive IFNαβ

**Figure 7:**
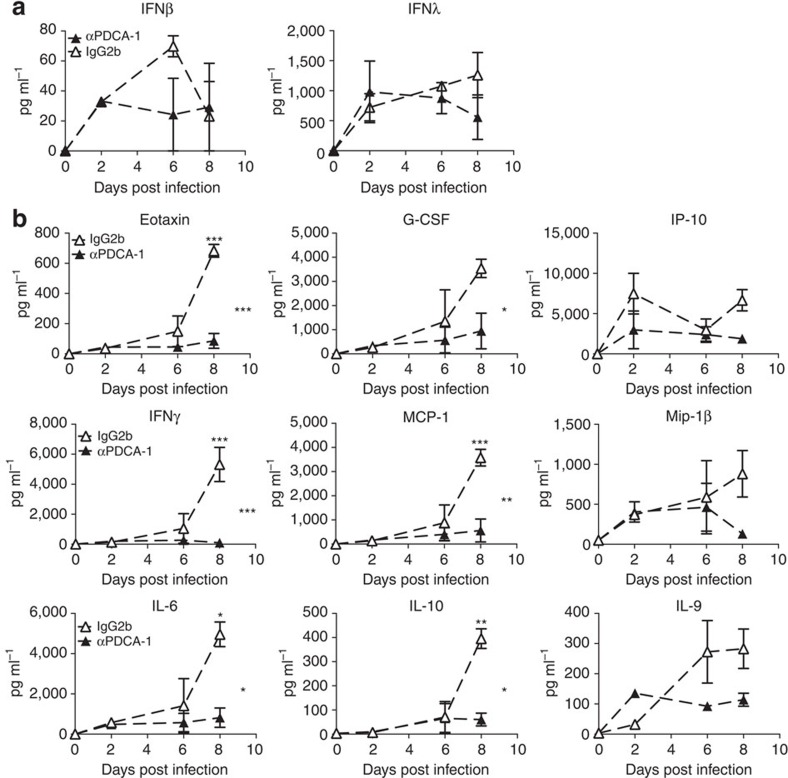
PDCA-1+ cell depletion reduces secretion of proinflammatory cytokines in infected 129S7 mice. (**a**,**b**) 129S7 mice were treated with αPDCA-1 (filled triangles) or isotype control (open triangles) and infected with X31. Cytokine concentrations in BAL fluid were measured (**a**) by ELISA for IFNβ and λ and (**b**) by Multiplex for indicated cytokines. Graphs show mean±s.e.m. where *n*=2–3 and ***P<0.0001, **P<0.001, *P<0.01 by two-way ANOVA with Bonferroni post tests. The symbols on the right of graphs indicate statistical significance of the whole curve, as tested by two-way ANOVA.

A principal source of systemic IFNαβ are plasmacytoid dendritic cells (pDCs)^31^, which have been demonstrated to be more abundant in naive 129 mice compared with other mouse strains^32^. We find higher frequencies and numbers of lung pDCs in 129 mice at all time points examined after infection, when compared with IFNαβR−/−(129) and B6 mice (Fig. 6a). Importantly, efficient depletion of PDCA-1+ cells including the entire pDC population (Fig. 6e and Supplementary Fig. 6) in 129 mice led to lower amounts of IFNα, a trend for less IFNβ and markedly decreased weight loss and morbidity while not affecting virus clearance (Figs 6b–e and 7a), demonstrating that morbidity was mediated by IFNαβ derived from pDCs or other cells upregulating PDCA-1 during infection.

Significantly, concentrations of the previously assessed pro-inflammatory cytokines were also significantly reduced in the BAL fluid of PDCA-1-depleted 129 mice, as compared with control mice (Fig. 7b). Pro-inflammatory cytokine levels found here are similar to cytokine and chemokine levels found in IFNαβR−/−(129) mice, indicating that IFNαβ derived from PDCA-1+ cells induces production of these inflammatory mediators. In contrast, IFNλ levels were unchanged on PDCA-1 depletion (Fig. 7a), suggesting that other cell types, possibly including lung epithelia, contribute to the production of this cytokine in the infected organism.

Frequencies and numbers of both inflammatory monocytes and NK cells (identified as shown in Supplementary Fig. 6) were reduced on PDCA-1 depletion (Fig. 6e), which may be due to a combination of direct depletion of monocytes and NK cells that upregulate PDCA-1 on infection and indirect effects of reduced cytokine levels. In addition, we obtained independent confirmation of the role of pDCs and monocytes when we used for depletion the mAb RB6-8C5, which is specific for the antigen Gr-1 that is composed of Ly6C expressed on pDCs and inflammatory monocytes and of Ly6G expressed on neutrophils. Treatment of 129 mice with this mAb-depleted pDCs, inflammatory monocytes and neutrophils and consequently resulted in a drastic reduction in influenza-related morbidity. Improved 129 resistance was not due to neutrophil depletion, as the neutrophil-specific mAb 1A8 did not change the course of disease (Fig. 6f). Treatment with anti-Asialo GM1 to deplete NK cells during influenza infection also failed to protect 129 mice against severe disease (Fig. 6g), indicating that NK cells alone do not mediate disease severity in influenza-infected 129 mice.

BM-derived Flt3-pDCs from 129 mice produced more IFNαβ and IFNλ than B6 pDCs when exposed to live influenza virus *in vitro* (Fig. 6h), confirming similar observations made with splenic pDCs treated with paraformaldehyde-inactivated virus^32^. Together these data suggest that 129 mice have a higher IFNαβ response through the combined effect of more pDCs at base line, higher recruitment into the lung on infection and higher responsiveness on a per-cell basis of the recruited pDCs and, potentially, other PDCA-1+ cells.

### IFNαβ-dependent expression of TRAIL and its ligand DR5

**Figure 8:**
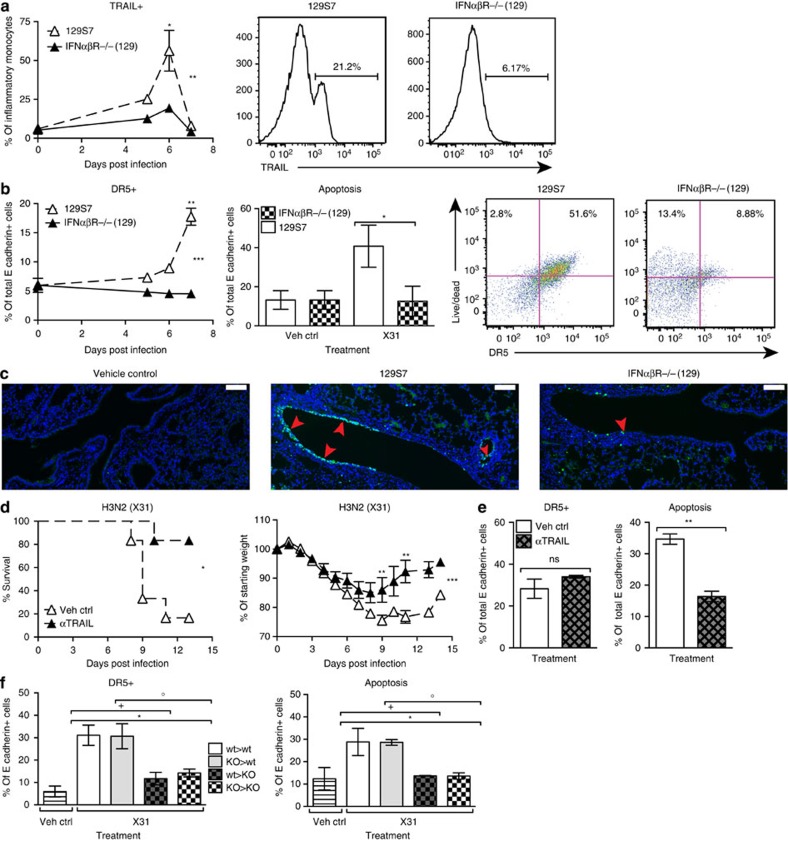
IFNαβ is upstream of TRAIL:DR5-mediated pathology in infected 129S7 mice. (**a**,**b**) 129S7 (open triangles) or IFNαβR−/−(129) (filled triangles) mice were infected intranasally with X31, and lung single-cell suspensions were prepared at the indicated time points after infection. At specified time points, flow cytrometric analysis was performed to assess expression of (**a**) TRAIL on inflammatory monocytes (which were gated as shown in Supplementary Fig. 6). Histograms show TRAIL expression on d6. (**b**) Flow cytrometric analysis of DR5 expression on epithelial cells (E cadherin+, CD45−) over time. At 7 d.p.i. epithelial cells were assessed for free amine staining as a measure of apoptosis. Dot plots show the correlation between free amine and DR5 stain on d7. (**c**) Lung sections from control and infected mice with the indicated genotypes were stained by TUNEL for apoptotic cells. Red arrowheads indicate TUNEL signal. Scale bar, 100 μm. (**d**,**e**) 129S7 mice were treated with the TRAIL-blocking mAb αCD253 (150 μg  per 200 μl i.p.) or with isotype control as indicated, mortality and morbidity were recorded throughout infection and (**e**) at 7 d.p.i. epithelial cells were assessed for DR5 expression and apoptosis. (**f**) BM chimeras were generated: 129S7>129S7 (wt>wt, open bars), IFNaβR−/−(129)>129S7 (KO>wt, grey bars), 129S7>IFNaβR−/−(129) (wt>KO, grey checked bars) and IFNaβR−/−(129)>IFNaβR−/−(129) (KO>KO, checked bars) and infected with X31, and expression of DR5 on epithelial cells and epithelial cell apoptosis was assessed at 7 d.p.i. Graphs show mean±s.e.m. and are representative of 2–3 independent experiments where *n*=3 for FACS data and *n*=6 for weight loss and survival. ***P<0.0001, **P<0.001, *P<0.01 by two-way ANOVA with Bonferroni post tests (TRAIL and DR5 expression and weight loss), Log-rank (Mantel–Cox) Test (survival) or Mann–Whitney test (DR5 expression and apoptosis) where * denotes wt>wt:KO>KO, +stands for KO>KO:wt>KO and ^○^ represents KO>KO:KO>wt. The symbols on the right of graphs indicate statistical significance of the whole curve, as tested by two-way ANOVA.

It has been proposed that, in severe influenza, interaction of TRAIL on macrophages with the TRAIL receptor DR5 on the epithelial cells leads to apoptosis of the latter cell population^33^. We therefore assessed levels of both molecules in IFNαβR−/−(129) and wt 129 mice and found increased expression of TRAIL on inflammatory monocytes and of DR5 on epithelial cells on infection in 129 wt mice compared with mice defective for IFNαβ signalling (Fig. 8a,b). Significantly, DR5 expression directly correlated with staining for cell apoptosis on wt but not on IFNαβR−/− lung epithelia cells (Fig. 8b). TUNEL staining on histological sections from infected 129 and IFNαβR−/− lungs also confirmed a higher incidence of epithelial cell apoptosis in wt mice (Fig. 8c).

To confirm that TRAIL and DR5 expressions require type I IFN and are not a function of disease burden, we also assessed expressions of TRAIL and DR5 on STAT1−/− inflammatory monocytes and epithelial cells. In spite of STAT1−/−(129) mice having a higher disease burden than their wt controls (Fig. 4c), STAT1−/− inflammatory monocytes and epithelial cells did not upregulate TRAIL or DR5 on their surface (Supplementary Fig. 7a). Furthermore, the low IFN-responding, resistant mouse strains B6 and BALB/c did upregulate TRAIL on monocytes and DR5 on epithelia, albeit not to the extent of the 129 strain (Supplementary Fig. 8a,b). Taken together, these data demonstrate that both TRAIL and DR5 expressions depend on type I IFN signalling and are not a result of severe disease burden.

Since TRAIL upregulation on CD8+ T cells, NK cells and pDCs has also been described^34^,^35^, we tested whether IFNαβ signalling is required for TRAIL expression on these cells. We find no difference in TRAIL levels on IFNαβR−/−(129) versus wt 129 CD8+ T cells, NK cells and pDCs, indicating that here type I IFNs are not required for TRAIL expression on these cells (Supplementary Fig. 9). In addition, the human apoptosis-inducing TRAIL receptors DR4 and DR5 are upregulated by influenza infection on the human alveolar epithelial cell line A549, demonstrating that this process is also observed in human cells (Supplementary Fig. 7b).

To test whether TRAIL/DR5 interaction contributes to epithelial cell apoptosis and morbidity of 129 mice, we blocked TRAIL using a blocking mAb in 129 mice and found that weight loss and morbidity were significantly reduced in mAb-treated mice (Fig. 8d). Consistent with this increased resistance to influenza-induced disease, anti-TRAIL-treated 129 mice had a significantly lower frequency of apoptotic airway epithelial cells at 7 d.p.i. However, anti-TRAIL treatment did not affect DR5 expression on epithelial cells (Fig. 8e). These results indicate that IFNαβ causes induction of TRAIL on monocytes recruited into the lung and of DR5 on lung epithelia, and that the interaction of these molecules leads to epithelial cell apoptosis. Blockage of this interaction protects from the severe disease observed in influenza-infected 129 mice.

To assess whether type I IFN signalling was required in epithelial cells for DR5 upregulation and for increased host morbidity, we generated bone marrow chimeras containing either IFNαβR-deficient stroma cells (wt>KO, KO>KO) or wt stroma cells (wt>wt, KO>wt). In these chimeras, epithelia insensitive to type I IFN signalling (wt>KO, KO>KO) did not upregulate DR5 expression in response to X31 infection. Conversely, chimeras generated in 129S7 hosts, where epithelial cells could sense IFNαβ signalling (wt>wt and KO>wt), showed an infection-induced increase in epithelial DR5 expression, and this correlated with increased epithelial apoptosis (Fig. 8f). Collectively, these data indicate that type I IFN signalling is required in epithelia for DR5 upregulation, leading to increased epithelial apoptosis.

Infection of the four types of chimeras with X31 revealed that susceptibility to influenza mediated pathology correlated with the ability of stromal cells such as epithelia to respond to type I IFN signalling (Supplementary Fig. 10a). To assess the type I IFN signal that induces DR5 expression on epithelial cells, we first measured levels of IFNα, -β and -λ in BAL fluid throughout X31 infection. wt>wt and KO>KO chimeras closely match the intact mice of the same genotype, while the wt>KO and KO>wt chimeras have intermediate levels of type I IFNs (Supplementary Fig. 10b). In the KO>wt chimeras, it is therefore unclear which cells are the source of IFNαβ and of the TRAIL-mediated signal required for epithelial apoptosis. To analyse these BM chimeras more in depth, we generated them using congenic wt 129.CD45.1 mice, to allow us to trace the origin of immune cells in the infected lung. In KO>CD45.1 wt chimeras, we find a residual population of CD45.1+ wt monocytes of host origin, and direct comparison between wt and IFNαβR−/− monocytes in these chimeras show that TRAIL levels are higher on the wt than on the KO cells, confirming that TRAIL upregulation on monocytes requires an IFNαβ signal (Supplementary Fig. 10d).

To understand whether the residual wt monocyte subset found in KO>wt chimera could also contribute to IFN production, we compared *in vitro* 129 and IFNAR−/−(129) BM-derived macrophages (BMDMs) in their ability to produce IFN in response to influenza exposure. We find that, like pDCs, 129 BMDMs produce more IFN than IFNAR−/−(129) BMDMs (Supplementary Fig. 10c), suggesting that KO>wt chimeras show intermediate IFN levels thanks to IFN production by residual wt host immune cells including monocytes. In conclusion, radioresistant host monocytes are the source both of IFNαβ and of TRAIL in KO>wt chimeras.

### Similar mechanisms of severe disease in DBA/1 and 129 mice

The correlation between high IFN levels and severe disease on influenza infection was found across a range of mouse strains (Fig. 1, Supplementary Figs 2 and 3). To test further whether the same underlying immune mechanisms lead to severe disease, we tested DBA/1 mice, which are described to be highly susceptible to influenza^36^. Similar to 129 mice, influenza-infected DBA/1 show high morbidity, high IFN type I and type III levels and high frequencies of lung pDCs, NK cells and inflammatory monocytes. In addition, TRAIL and DR5 levels are high on monocytes and epithelial cells, respectively, and epithelial apoptosis is increased compared with resistant B6 mice (Supplementary Fig. 11a–c). Importantly, reducing inflammation by administration of a mAb directed against Gr-1, which depletes pDCs and monocytes and improves disease in 129 mice (Fig. 6f), also rescues DBA/1 mice from severe disease (Supplementary Fig. 11d). Similarly, blocking TRAIL rescues DBA/1 mice (Supplementary Fig. 11e) as it does with 129 mice (Fig. 8d). We conclude that the IFNαβ-driven inflammation leading to severe disease is a general phenomenon in action across a wide range of mouse models of influenza.

## Discussion

Here, we demonstrate that host-intrinsic differences can determine the outcome of influenza infection and that IFNαβ is a host-specific determinant with a dose-dependent detrimental potential. More susceptible mouse strains produce markedly higher levels of IFNαβ in response to influenza infection than resistant strains. Detailed comparison of susceptible 129 with resistant B6 mice allows us to delineate a disease pathway leading from high numbers of hyper-reactive pDCs producing excessive IFNαβ amounts sustained over time, which in turn causes uncontrolled inflammation and lung epithelial damage mediated by TRAIL–DR5 interaction.

Here we identify IFNαβ as a host factor that directly mediates inflammation, morbidity and mortality in an acute viral infection. Our findings are, however, reminiscent of infection by HIV or SIV. Higher and more sustained IFN responses are detected in pathogenic SIV infection in rhesus macaques, while natural hosts without disease progression show lower IFN levels^37^,^38^. Similar data were found in humans when rapid progressors were compared with viremic nonprogressors^39^. Interestingly, the expression of IFNα in pDCs and of TRAIL and DR5 in tonsil tissue was found to be higher in HIV progressors compared with long-term nonprogressors^40^. Furthermore, IFNαβ has recently been demonstrated to contribute to the establishment and maintenance of chronic lymphocytic choriomeningitis virus infection in mice, where an elevated type I IFN response correlated to increased expression of immunosuppressive genes leading to an impaired adaptive immune response^41^,^42^. In contrast, we show here that in acute influenza infection, IFNαβ contributes to morbidity and mortality through excessive innate immune responses leading to tissue damage.

Modern histopathological analysis of autopsy samples from human H1N1 1918 influenza infection revealed massive lung damage involving significant destruction of the respiratory epithelium in severe influenza^43^. Here we demonstrate that IFNαβ-mediated DR5 upregulation on lung epithelial cells resulted in epithelial cell apoptosis and therefore host morbidity. IFNαβR deficiency in 129 epithelia or blockade of TRAIL/DR5 interaction protected mice against destruction of respiratory epithelium. By identifying TRAIL and DR5 interaction as a downstream mediator of IFNαβ-dependent morbidity and mortality in influenza infection, we provide a molecular mechanism that links high IFNαβ levels to epithelial cell death and therefore high susceptibility. Our data are in agreement with previous studies showing an involvement of TRAIL in severe forms of influenza^33^ and report on the IFN dependence of TRAIL upregulation on monocytes^44^. Our data demonstrate *in vivo* that IFNαβ signalling is specifically required in epithelial cells for DR5 upregulation, which leads to epithelial apoptosis and eventually to host morbidity. HIV progression has also been linked to IFNαβ-dependent, TRAIL-mediated death of virus-infected CD4 T cells^28^. An alternative mechanism was described by van Grevenynghe *et al.*^45^ who show TRAIL-mediated B-cell apoptosis in HIV infection due to disrupted IL-2 signalling. As similar observations were made in hepatitis C infection^46^, a common theme appears to emerge that one pathway of immunopathology in virus infection may be excessive apoptosis induction in virus-infected cells downstream of high levels of IFNαβ and TRAIL.

IFNαβ has well-documented antiviral effects. Our finding that lack of IFNαβ signalling does not have deleterious consequences for virus control is most likely explained by the redundancy between IFNαβ and other cytokines including IFNλ^12^. Although devoid of type I IFN signalling, 129 IFNαβR−/− mice exhibit IFNλ concentrations that are comparable to resistant B6 mice during infection. The similar levels of IFNλ in both resistant genotypes may therefore be sufficient to keep influenza virus replication under control. In fact, the induction of ISGs with antiviral function was comparable in infected airway epithelia from IFNαβR−/− and parental 129 mice. In contrast, influenza infection did not induce ISGs in STAT1−/− epithelia, and STAT1−/−(129) mice were even more susceptible to influenza infection than 129 wt mice. These results are in agreement with previous studies using STAT1−/−(129) mice^13^. In conclusion, comparison between wt, IFNαβR−/− and STAT1−/− genotypes in a high IFN producer genetic background such as 129 allowed us to unmask the pathogenic potential of IFNαβ and its nonredundant role in causing excessive inflammation in response to influenza infection. In contrast, we show that IFNαβ is redundant for the induction of antiviral ISGs at the epithelial sites of influenza replication.

Results contrasting our own were obtained when IFNαβR−/−(129) mice were infected with the influenza strain WSN and were shown to be more susceptible than wt 129 mice^13^. However, the WSN strain shows an unusual neurotropism that might explain this apparent discrepancy: if infected neurons cannot rely on IFNλ as a back up system to induce an antiviral state, then IFNαβR deficiency may have a great impact on viral control in these cells, leading to increased susceptibility to WSN infection. Since influenza virus usually infects preferentially airway epithelia and IFNαβ is redundant for ISG induction at this site, blockade of IFNαβ signalling will change the outcome of disease through modulation of the nonredundant, immunostimulatory IFNαβ effects. While our data place IFNαβ upstream of the cytokine storm associated with severe disease in 129 mice, it will be interesting to determine the downstream cell type that is triggered by IFNαβ to produce these high levels of inflammatory cytokines. Candidates include immune cells and endothelial cells, both of which are potential cytokine sources in influenza infection^47^.

When infections with low- or high-pathogenicity viruses are used to compare mild with severe influenza, high production of proinflammatory cytokines is mostly associated with severity, and immune-mediated pathology is one of the suggested mechanisms^1^,^48^. In contrast to those cytokines, IFNαβ induction correlates sometimes directly and sometimes inversely with the pathogenicity of the virus^49^,^50^,^51^,^52^. These comparisons do not allow the distinction between virus-specific and host-specific factors contributing to disease severity. Back-crosses between influenza-susceptible and -resistant mouse strains show higher levels of proinflammatory cytokines in susceptible strains^53^. Here we demonstrate that in response to infection by the same amount of the same strain of influenza virus, and in the face of identical amounts of virus detected in the lung throughout the early phase of infection, IFN levels diverge early on in the response depending on the mouse strain background, showing that host-specific factors are important to determine the magnitude of the IFN response. One of these factors appears to be the steady-state and influenza-induced number of pDCs present in the host.

Genetic loci determining absolute numbers and frequencies of pDCs have been recently identified, confirming that the size of the pDC compartment is a genetic trait^54^. Previous studies have assessed the role of pDCs in influenza infection. Since most of these studies were performed in BALB/c or B6 backgrounds where low levels of IFNαβ appear to be protective, these studies did not find effects similar to the results presented here^55^,^56^. In contrast, no studies had been performed so far in 129 mice known to contain high numbers of pDCs. Interestingly, one study on lethal influenza shows that pDCs contribute to mortality through Fas:FasL-mediated elimination of CD8 T cells^57^; however, no involvement of IFNαβ was indicated in that report. Although a similar mechanism may contribute to mortality in 129 mice, we found increased mortality of 129 compared with B6 mice also on a Rag-deficient background, and we did not find differences between B6, 129 and IFNαβR−/−(129) mice in the numbers or activation phenotype of influenza-specific CD8 T cells as identified by MHC class I tetramer staining. This further indicates that another suggested mechanism linking TRAIL expression on CD8 T cells to the control of the CD8 response^35^ is not the mechanism at work here. We find no differences in CD8 TRAIL expression between the different mouse genotypes under study here and strain differences in susceptibility are also found in Rag−/− mice devoid of CD8 T cells.

The protective potential of exogenous IFNα or β has been tested in animal models. In all those studies, animals were treated before infection, with the aim of inducing an antiviral state in cells before they were infected. This situation is different to high IFN induction during infection as reported here, where IFN is a consequence of viral infection and does not precede it. Even in infection models of a natural host for influenza, the ferret, pretreatment with IFNα had minor effects that often lasted only 1 day^58^. Another study demonstrated that oral application of high IFN doses to mice did not improve influenza-induced disease, while low dose regimens did^59^, findings that are in line with our data. We also observe that IFNα treatment post infection leads to higher morbidity of B6 mice and renders their disease phenotype similar to that of susceptible 129 mice with endogenously high IFN responses. However, we believe that influenza-induced IFNαβ levels are not the only host difference between the resistant and susceptible strains assessed here, as the host background also determines what effect IFN can have. This was evident in studies where IFNα pretreatment of Mx1+/+ mice helped control a high-pathogenicity influenza strain, while pretreatment had no effect on Mx1−/− mice^60^.

Interferon induces a transcriptional response through autocrine, paracrine or systemic effects. Among the hundreds of proteins induced by interferon, an anti-influenza effect was shown for a subgroup including the Mx gene products, PKR, and the families of IFITM, IFIT and RNase L proteins^61^. IFITM3 was recently shown to be relevant to anti-influenza resistance not only in mice but also in humans, and they appear to function similarly in both species^3^, suggesting that IFITM3 is an important viral restriction factor conserved through evolution. The Mx1 gene product in outbred mice has been shown to have strong antiviral effects on some influenza strains^62^, while other strains, including the pandemic H1N1 strain used in this study, are resistant to Mx effects, as the presence or absence of the Mx gene did not alter replication of these virus strains *in vitro* and *in vivo*^63^,^64^. The human Mx homologue is less efficient in its antiviral function *in vivo*^65^, perhaps reflecting the different antiviral mechanisms used by human and mouse Mx1 gene products or viral adaptation to evade human Mx effects, and no genetic study in humans has linked the existing polymorphisms in the human Mx genes to altered susceptibility^4^.

A group of subjects with increased IFN induction on influenza virus exposure and stronger responses to IFNαβ are individuals with Down’s syndrome who carry three copies of the human chromosome 21 containing genes encoding the IFNαβR, and, interestingly, MxA and MxB. Trisomic blood cells show strongly increased IFN responses to influenza virus^66^, and trisomic macrophages show higher sensitivity to IFN^67^. Intriguingly, these individuals are reported to have a higher incidence of respiratory infections including seasonal and pandemic influenza and a higher risk of influenza-related severe disease^68^,^69^, therefore representing a patient group where heightened IFN levels and responsiveness are linked to higher influenza severity.

In conclusion, we show here through genetic, cell ablation and mAb-blocking experiments that in influenza-infected hosts, excessive amounts of IFNαβ produced by PDCA-1+ cells are upstream of a proinflammatory, pathogenic mechanism culminating in high morbidity and mortality mediated by TRAIL–DR5 interaction. In the human population, the at-risk group for severe influenza may contain individuals with high frequencies of pDCs or a propensity to strong IFN responses. Our observations therefore have important implications for prediction of susceptibility to severe influenza and for treatment of disease induced by this infection.

## Methods

### Mice

129/SvEvBrd-Hprt^b-m2^ mice and IFN type I receptor α-chain-deficient (IFNαβR−/−) generated on the 129SvEv background^11^ were purchased from B&K Universal. Identity of the genetic background between these strains was confirmed by SNP and microsatellite analysis (Charles River). CBA/J mice were kindly provided by Dr A. O’Garra (MRC-NIMR). Recombination activating gene-2-deficient (Rag-2−/−) mice on the 129Sv background were kindly provided by Dr F. Powrie (Univ. of Oxford). The above mice, 129S8 mice, BALB/c, C57BL/6 mice and Rag-1−/− mice on the C57BL/6 background were bred at the MRC-National Institute for Medical Research under specific pathogen-free conditions. DBA/1 and 129 × 1/SvJ mice were purchased from Jackson Laboratory, DBA/2 mice from Harlan, 129S6/SvEv-Stat1^tm1Rds^ (Stat1−/−), 129S5 and 129S6 from Taconic, and kept in specific pathogen-free isolators until use for experiments. Unless otherwise stated, experiments were performed on 6- to 12-week-old male mice. Clinical symptoms during influenza infection were scored based on presentation of piloerection, hunched posture, labored breathing, swaying gait, hypothermia and reduced spontaneous or provoked movement, with each symptom scoring as 1. All protocols for breeding and experiments with animals were approved by the local ethical committee of the MRC-NIMR and are part of a project license approved by the Home Office, UK, under the Animals (Scientific Procedures) Act 1986.

### Influenza viruses

A/PR/8/34 (PR8, H1N1), X31 (a H3N2 reassortant with PR8 backbone) and A/California/04/09 (Cal09, H1N1) (kind gifts from Dr J. Skehel, MRC-NIMR) were grown in the allantoic cavity of 10 day-embryonated hen’s eggs and were free of bacterial, mycoplasma and endotoxin contamination. Alternatively, virus was grown in Madin-Darby Canine Kidney (MDCK) cells, a kind gift from Dr J. McCauley, MRC-NIMR. All viruses were stored at −80 °C and titrated on MDCK cells. Mice were anaesthetised by inhalation with isofluorane and infected via the intranasal (i.n.) route with 30μl of indicated influenza strain diluted in PBS. Virus was quantified in infected lungs by qPCR on cDNA from whole lungs for the Matrix gene:


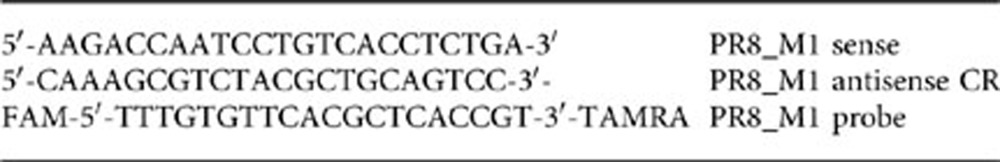


Alternatively, virus was titrated on MDCK cells, and the 50% tissue culture infective dose (TCID_50_) was determined by eight replicates of 10-fold serial dilutions using the Spearman and Faerber fit.

### Treatment of mice

C57BL/6 mice were infected with X31 (8,000 TCID_50_ per 30μl) or inoculated with Vehicle control i.n., then treated with Recombinant Mouse IFNα4 (PBL), 3.5 × 10^4^IU per 200 μl or Vehicle Control via the intraperitoneal (i.p.) route on days 1–6 post infection. To specifically deplete pDCs, 129 mice were treated with αPDCA-1 (Cambridge Bioscience) or IgG2b isotype-matched control, 500 μg  per 200 μl i.p. on day 1 of infection with X31: 800 TCID_50_, and every 48 h thereafter. 129 mice were treated with the Gr-1 reactive RB6-8C5, Ly6G reactive 1A8 or Isotype control (IgG2b) (500 μg  per 200 μl i.p.) on day 1 of infection and repeated every 48 h throughout X31 (800 TCID_50_) infection. To deplete NK cells, 129 mice were treated with rabbit αAsialo GM1 serum (200 μl) on days 1, 3 and 7 post X31 infection. All depletions were confirmed by flow cytometry. To block TRAIL action, 129 mice were treated i.p. with 150 μg  per 200 μl of αCD253 (N2B2) (Cambridge Bioscience) or isotype control (IgG2a) every 24 h on days 0–9 post infection with 800 TCID_50_ of X31.

### *In vitro* stimulation of pDCs and macrophages

129, IFNαβR−/−(129) or C57BL/6 bone marrow cells were obtained by flushing femurs and tibias with RPMI-1640 (BioWhittaker), using a 23 gauge needle. Red blood cells were lysed using ammonium chloride, and cells were cultured in Flt3 supplemented (100 ng ml^−1^) (Pepro Tech) for pDCs or, for macrophages, L cell sup supplemented (10%) (a kind gift from Anne O’Garra, MRC-NIMR) culture media (10% fetal calf serum (PAA), L-glutamine, penicillin, streptomycin, and β-mercaptoethanol in RPMI-1640). Media was replaced at day 4 of macrophage cultures, which were harvested at day 7 by collection of the adherent cells. Culture was found to contain 95% macrophages, identified as FSC^hi^, SSC^hi^, F4/80^+^, CD11b^+^ by flow cytometry. For pDCs, media was replenished at days 3 and 6 of culture, and cells were harvested at day 9. Harvested cells were preincubated with Fc block and biotin-conjugated B220 in 2% BSA (PBS) before a 30-min incubation with anti-biotin conjugated magnetic beads. pDCs were then positively selected using an LS Columns and the QuadroMACS separator, as per manufacturer’s instructions (Miltenyi Biotech) and found to be 95% pure based on FSC^lo^, SSC^lo^, αPDCA-1^+^, Siglec-H^+^, CD11b^−^ CD11c^int^ as analysed by flow cytometry. pDCs were seeded at 6 × 10^4^ cells per well, macrophages at 2 × 10^5^ cells per well and rested for 24 h before stimulation with X31 multiplicity of infection (MOI) of 1 or vehicle control for 24 h. Supernatants were then collected and stored at −70 °C until samples were analysed.

### RNA extraction

Whole lungs were collected in TRIzol (Invitrogen) and homogenized using Polytron PT 10–35 GT (Kinematica). MTEC cultures were lysed directly in the transwells, using the Qiagen RNeasy mini kit, according to the manufacturer’s instructions. Total RNA was prepared using phenol/chloroform extraction, and cDNA was generated from these samples using Thermoscipt RT–PCR system, as per manufacturer’s instructions (Invitrogen). The cDNA served as a template for the amplification of genes of interest and the housekeeping gene (Hprt1) by real-time PCR, using TaqMan Gene Expression Assays (Applied Biosystems), universal PCR Master Mix (Applied Biosystems) and the ABI-PRISM 7900 sequence detection system (Applied Biosystems). The fold increase in mRNA expression was determined using the ΔΔCt method relatively to the values in mock-treated samples, after normalization to Hprt1 gene expression.

### Microarray data analysis

Lungs were homogenized in TRI Reagent (RiboPure kit, Ambion), and total RNA isolated according to manufacturer’s instruction. RNA was hybridized to Illumina.SingleColor.Mouse WG-6_V2_0_R0_1127 microarrays. Raw data were processed using GeneSpring GX version 11.5 (Agilent Technologies). After background subtraction each probe was attributed a flag to denote its signal intensity detection *P*-value. Flags were used to filter out probe sets that did not result in a ‘present’ or ‘marginal’ call in at least 50% of the samples, in any one out of six experimental conditions. Next, a per-gene normalization was applied by dividing each messenger RNA transcript by the median intensity of mock-infected samples. All statistical analysis was performed after this stage: a two-way ANOVA (parameters: treatment and genotype) was performed to identify gene significantly differentially expressed relative to controls (≥fourfold change; *P*<0.01, Benjamini-Hochberg multiple test correction).

### Protein analysis

BAL fluid was recovered from naive and infected mice, centrifuged at 1,300 r.p.m., 5 min at 4 °C and supernatant collected. X31-stimulated pDC supernatants were collected after 24 h stimulation. Concentrations of IFNα, β (PBL Biomedical Laboratories) and λ (R&D) were measured by ELISA as per the manufacturer’s instructions. Concentrations of Eotaxin, G-CSF, IFNγ, MCP-1, IP-10, Mip-1β, IL10, IL-9 and IL-6 were assessed by using Milliplex Map Kit (Millipore) as per the manufacturer’s instructions and read on a Luminex 100 (BioRad).

### Microneutralization assay

Neutralizing antibodies in serum were assessed by using a microneutralization assay. In brief, serum samples were heat inactivated for 30 min at 56 °C, diluted 1:100 then serially diluted 1:3 in duplicate in 96-well flat-bottomed plates. Serum dilutions were preincubated with X31, 300 TCID_50_ per well for 1 h at 37 °C then added to MDCK cells and incubated for a further 22 h at 37 °C. After incubation, cells were washed and fixed, and neutralization capability was then assessed by staining for FITC-conjugated influenza nucleoprotein (Oxoid) and detected with a horseradish peroxidase-conjugated anti-FITC antibody (Roche). The reaction was then developed with tetramethylbenzidine substrate (eBioscience) for 15 min, stopped using H_2_SO_4_, and absorbance was read at 450 nm using a Safire2 reader (Tecan).

### Flow cytometry

Leukocytes from the lung were enumerated using flow cytometry. In brief, lungs were excised from naive and infected mice and homogenized using gentleMACS (Miltenyi), as per the manufacturer’s instructions. Lungs were then passed through a 70-μM cell strainer and washed with FACS buffer (10% BSA in PBS Azide). Red blood cells were lysed using ammonium chloride, and cells were seeded into a 96-well U-bottom plate at 1 × 10^6^ per well. Cells were preincubated with anti-FcγRIII/II (Fc block) in FACS buffer before a 30-min incubation with one or more of the following fluorochrome-labelled antibodies (used at a dilution of 1:200 and purchased from Cambridge Bioscience, unless otherwise stated): FITC-conjugated αPDCA-1 (Clone: 927, dilution 1:2,000) (Dendritics), FITC-conjugated NKp46 (Clone: 29A1.4), FITC-conjugated E-Cadherin (Clone: 36/E-Cadherin) (BD Pharmingen), FITC-conjugated Ly6C (Clone: HK1.4), FITC-conjugated NP_366–374_ H-2D^b^ tetramer (a kind gift from Carmela De Santo and Vincenzo Cerundolo, University of Oxford) PE-conjugated Siglec-H (Clone: 551), PE-conjugated TRAIL (Clone: N2B2), PE-conjugated DR5 (Clone: MD5-1), PerCP Cy5.5-conjugated Ly6C (Clone: HK1.4, dilution 1:2,000), PE Cy7-conjugated CD11b (Clone: M1/70, dilution 1:4,000), APC-conjugated CD45 (Clone: 30-F11), APC-conjugated F4/80 (Clone: BM8, dilution 1:100), APC Cy7-conjugated Ly6G (Clone: 1A8), APC Cy7-conjugated CD3 (Clone: 17A2), BD Horizon V450-conjugated CD11c (Clone: HL3) (BD bioscience). Cells were then washed with PBS x2 and counter stained with LIVE/DEAD Fixable Dead Cell Stain (Life Technologies) and, or 7AAD (Life Technologies) to enumerate apoptotic and dead cells (respectively) then analysed using a LSR II (Becton Dickinson).

### Histology

Whole lungs were perfused with 10% neutral buffered formaldehyde (NBF) *in situ*. Tissue was then fixed overnight in 10% NBF, embedded in paraffin and sectioned. Lung specimens were stained with haematoxylin and eosin (H&E) and then subjected to gross and microscopic pathologic analysis. For TUNEL staining, slides were deparaffinized and stained for apoptotic cells using ApopTag Fluorescein *In Situ* Apoptosis Detection Kit (Miltenyi) as per the manufacturer’s instructions.

### Primary mouse tracheal epithelial cell culture

Isolation and culture of primary mouse tracheal epithelial cell culture (MTEC) were performed as follows^70^: in brief, cells isolated by enzymatic treatment were seeded onto a 0.4-μm pore size clear polyester membrane (Corning) coated with a collagen solution. At confluence, media was removed from the upper chamber to establish an air-liquid interface (ALI). Fully differentiated, 10- to 14-day-old post ALI cultures were routinely used for experiments.

### A549 cell line

Human A549 cells (a kind gift from Dr J. McCauley, MRC-NIMR) were maintained in Dulbecco’s modified Eagle’s medium-high glucose, (DMEM GlutaMAX Supplement) (gibco-Life technologies) supplemented with 5% fetal calf serum (FCS). A549 cells were seeded on to 24-well plates and when confluent, used for experiments.

### Statistical analysis

Data shown as the means ±s.e.m. Sample sizes were designed to give statistical power, while minimizing animal use. Data sets were analysed by two-way ANOVA with Bonferroni post tests (weight and cytokine concentration time courses) Log-rank (Mantel-Cox) Test (survival) and Mann–Whitney Test. GraphPad Prism 5 (GraphPad Software, San Diego, CA) was used for data analysis and preparation of all graphs. *P*-values less than 0.01 were considered to be statistically significant.

## Additional information

**Accession codes:** Microarray data have been deposited in Gene Expression Omnibus database under accession code GSE55403 for the superseries.

**How to cite this article:** Davidson, S. *et al.* Pathogenic potential of interferonαβ in acute influenza infection. *Nat. Commun.* 5:3864 doi: 10.1038/ncomms4864 (2014).

## Supplementary Material

Supplementary Information Supplementary Figures 1-11
