# Supplementary material for: Pathogenic potential of interferon αβ in acute influenza infection
Source: Nat Commun. 2014 May 21;5:3864. doi: 10.1038/ncomms4864 (PMC4033792; doi:10.1038/ncomms4864)
Supplement: Supplementary Information — Supplementary Figures 1-11 [file ncomms4864-s1.pdf]

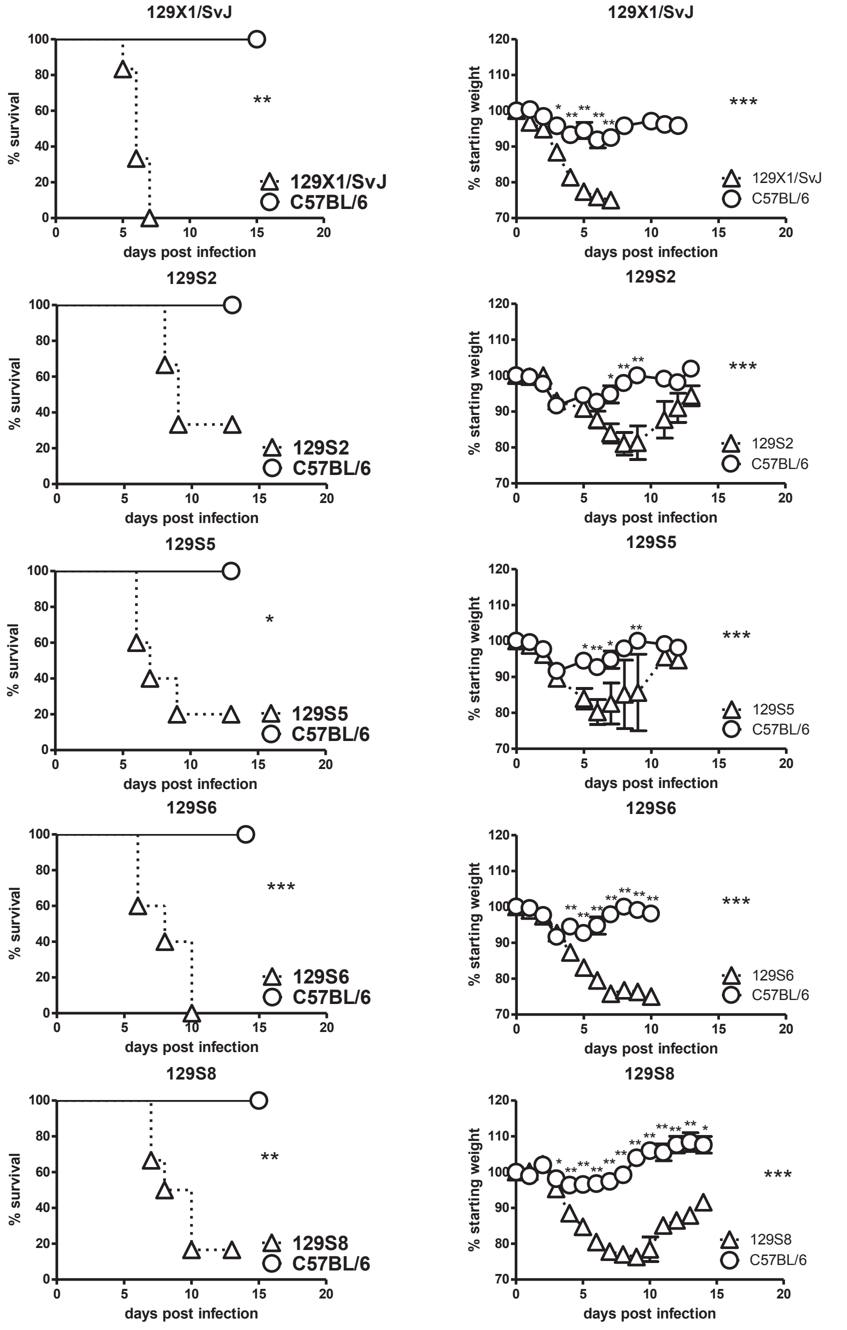

Supplementary Figure 1: All 129 substrains tested demonstrate higher susceptibility to X31 induced disease as compared to the B6 strain.

Indicated 129 substrains (triangles) or B6 (circles) mice were infected i.n with X31 (800 TCID50) and weight loss and mortality were recorded throughout infection. Graphs show mean ± s.e.m, n=5-6, ***p<0.0001, **p<0.001, *p<0.01 by 2-way ANOVA with Bonferroni post tests (weight loss) or Log-rank (Mantel-Cox) Test (survival).

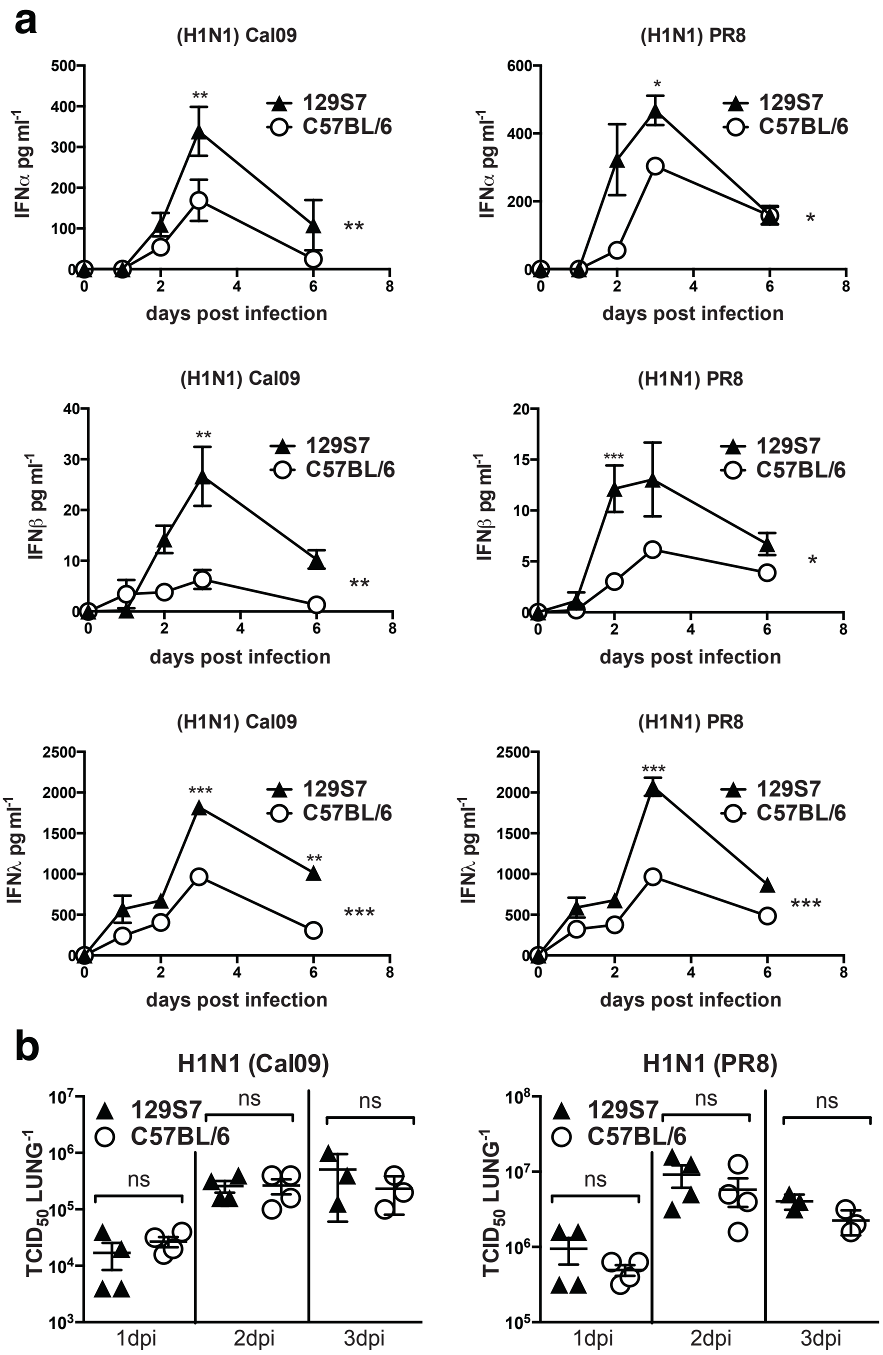

Supplementary Figure 2: Increased susceptibility of 129S7 mice to influenza-induced disease correlates with higher BAL concentrations of IFNα, β and λ, despite comparable viral load. 129S7 (filled triangles) or B6 (open circles) mice were infected i.n with either Cal09 or PR8, **(a)** concentrations of IFNα, β and λ in BAL fluid were measured by ELISA and **(b)** virus titers in lung homogenates were measured by TCID50 determination on MDCK cells. Graphs show mean ± s.e.m and are representative of 2 independent experiments where n=3-4. ***p<0.0001, **p<0.001, *p<0.01 by 2-way ANOVA with Bonferroni post tests (ELISA) or Mann Whitney test (viral quantification).

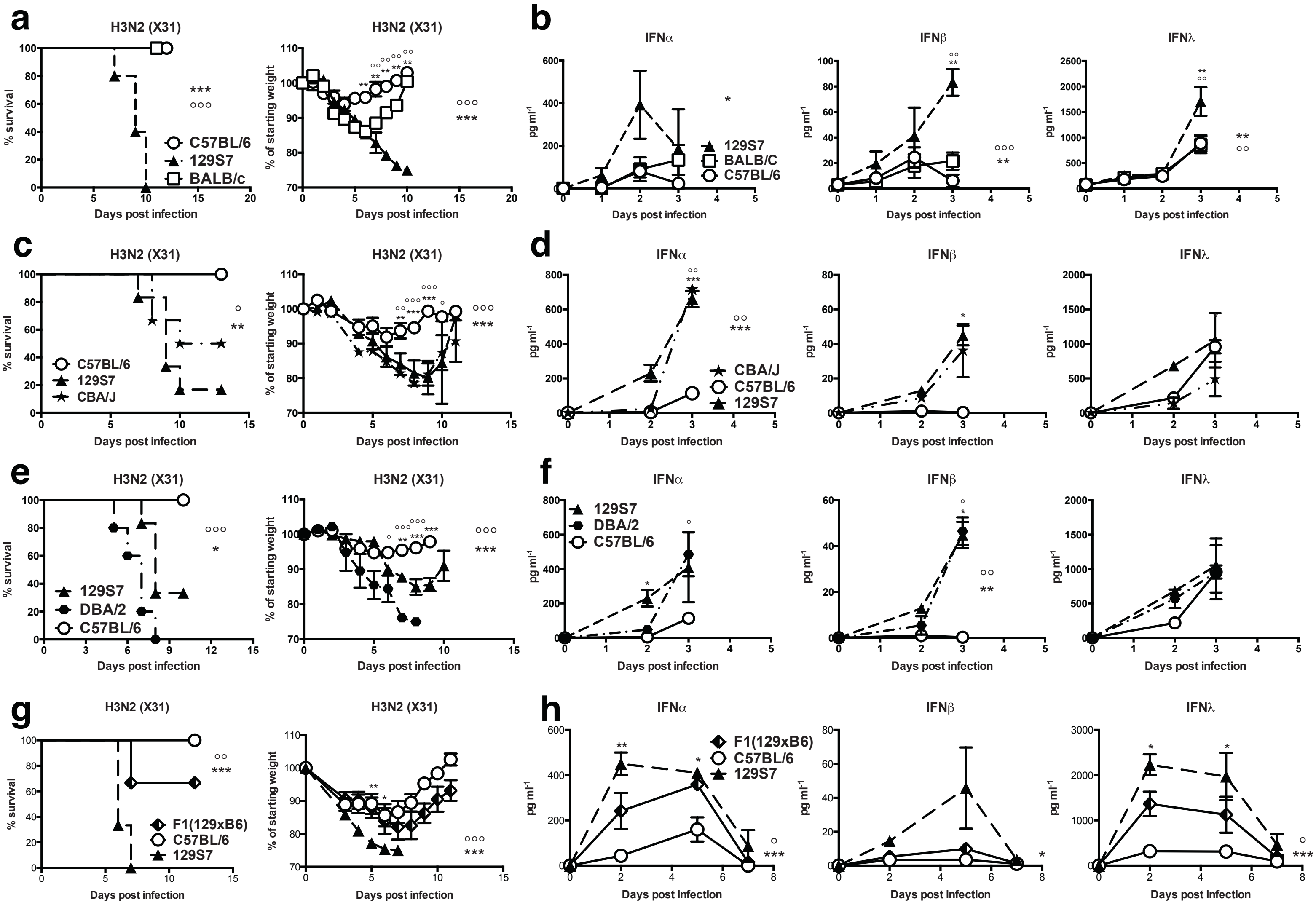

Supplementary Figure 3: Mouse strain susceptibility to influenza induced disease correlates to high type I IFN concentrations in BAL fluid

129S7, C57BL/6, BALB/C, CBA/J, DBA/2 and F1(129xB6) mice were infected with X31: **(a, b, g, h)** 8000 $TCID_{50}$ or **(c-f)** 800$TCID_{50}$. **(a, c, e, g)** Weight loss and mortality were recorded and **(b, d, f, h)** IFN concentrations BAL measured by ELISA. Graphs show mean ± s.e.m, where n=6 for weight loss and survival and n=2-3 for ELISA data. B6:129S7 *, BALB/C:129S7 ○, B6:CBA/J ○ and B6:DBA/2 ○, F1(129xB6):129S7 ○ where *** p<0.0001, ** p<0.001, * p<0.01 by 2-way ANOVA with Bonferroni post tests (weight loss and ELISA) or Log-rank (Mantel-Cox) Test (survival).

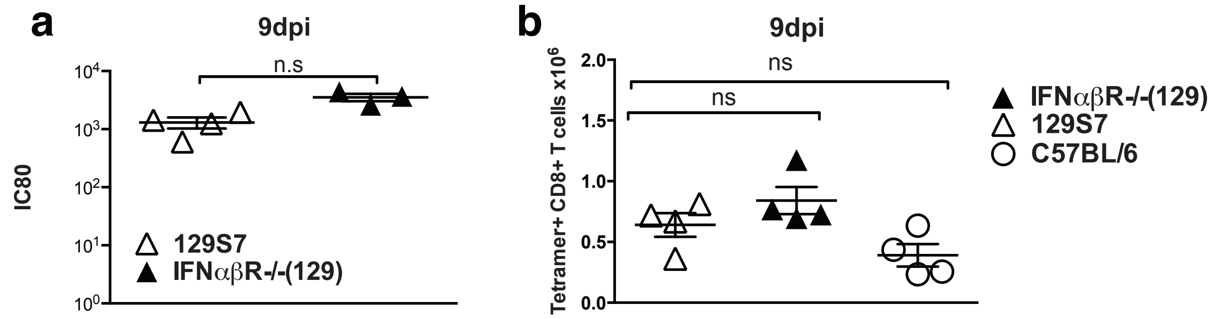

Supplementary Figure 4: The X31 specific adaptive response is comparable in 129 and IFNαβR-/-(129) mice.

129 (open triangles), IFNαβR-/- (129) (filled triangles) and C57BL/6 mice (open cirlces) were infected with X31. **(a)** At 9dpi sera were taken from indicated mouse strains and X31 neutralising antibody titre was measured by microneutralisation assay. **(b)** Lung single cell suspensions were prepared at 9dpi and flow cytometric quantification of influenza specific $NP_{366–374}$ H-2D^b tetramer+ CD8+ T cells (CD8+, CD3+, CD4-, FSClo, SSClo) was performed. Significance tested by Mann Whitney test.

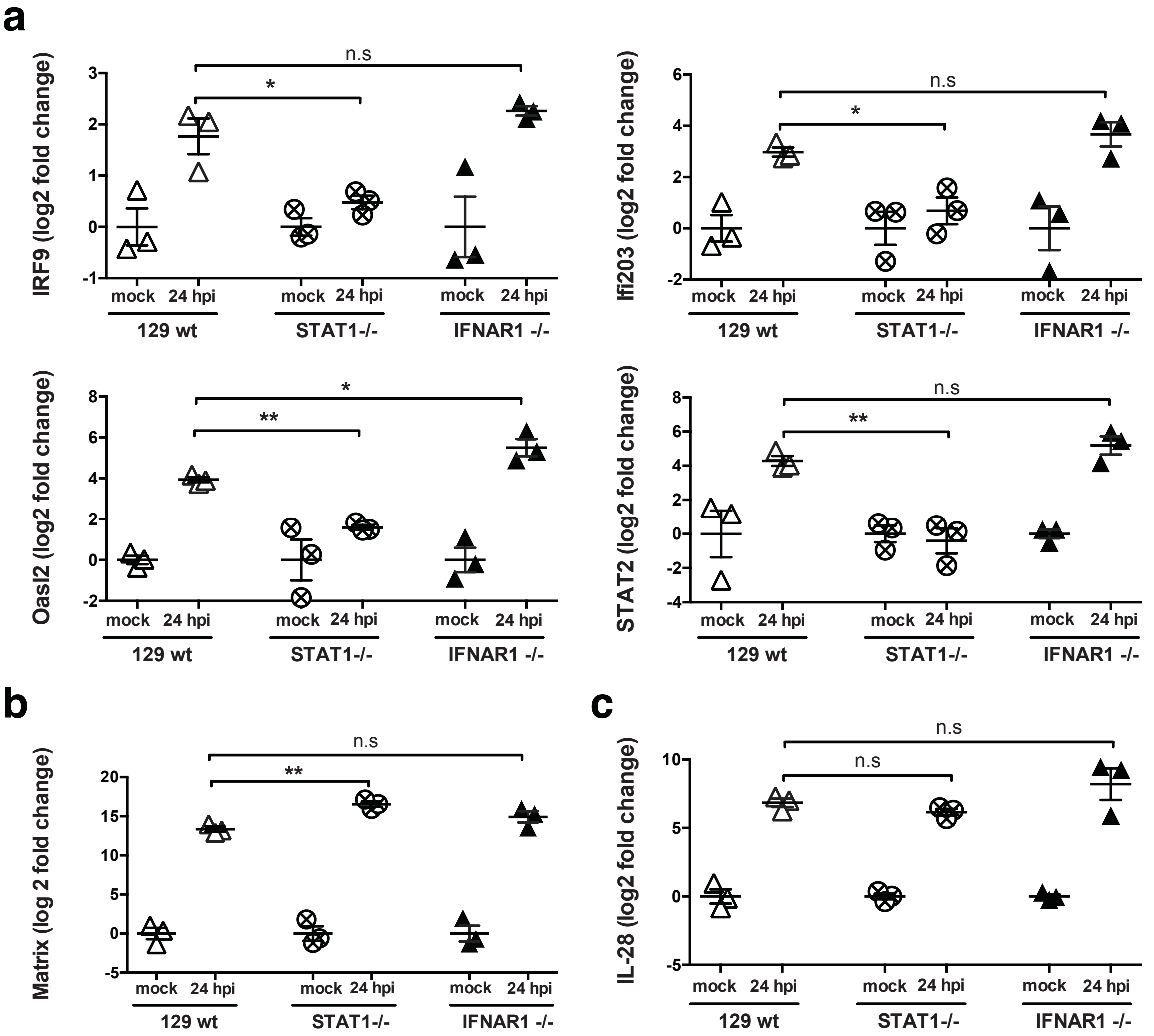

Supplementary Figure 5: Types I and III IFNs are redundant, only STAT1 is required for ISG induction and virus control upon influenza.

(a, b, c) STAT1-/-(129), IFNαβR-/-(129) and 129 epithelial cell cultures were infected with MDCK grown PR8 at a MOI of 1. At 24hrs post infection up regulation of **(a)** Oasl2, STAT2, IRF9, Ifi203, **(c)** IL-28 (IFNλ) mRNA and **(b)** replication of PR8 was assessed by RT-PCR. Graphs show mean ± s.e.m and are representative of 2 independent experiments where n=3, significance tested by Mann Whitney test.

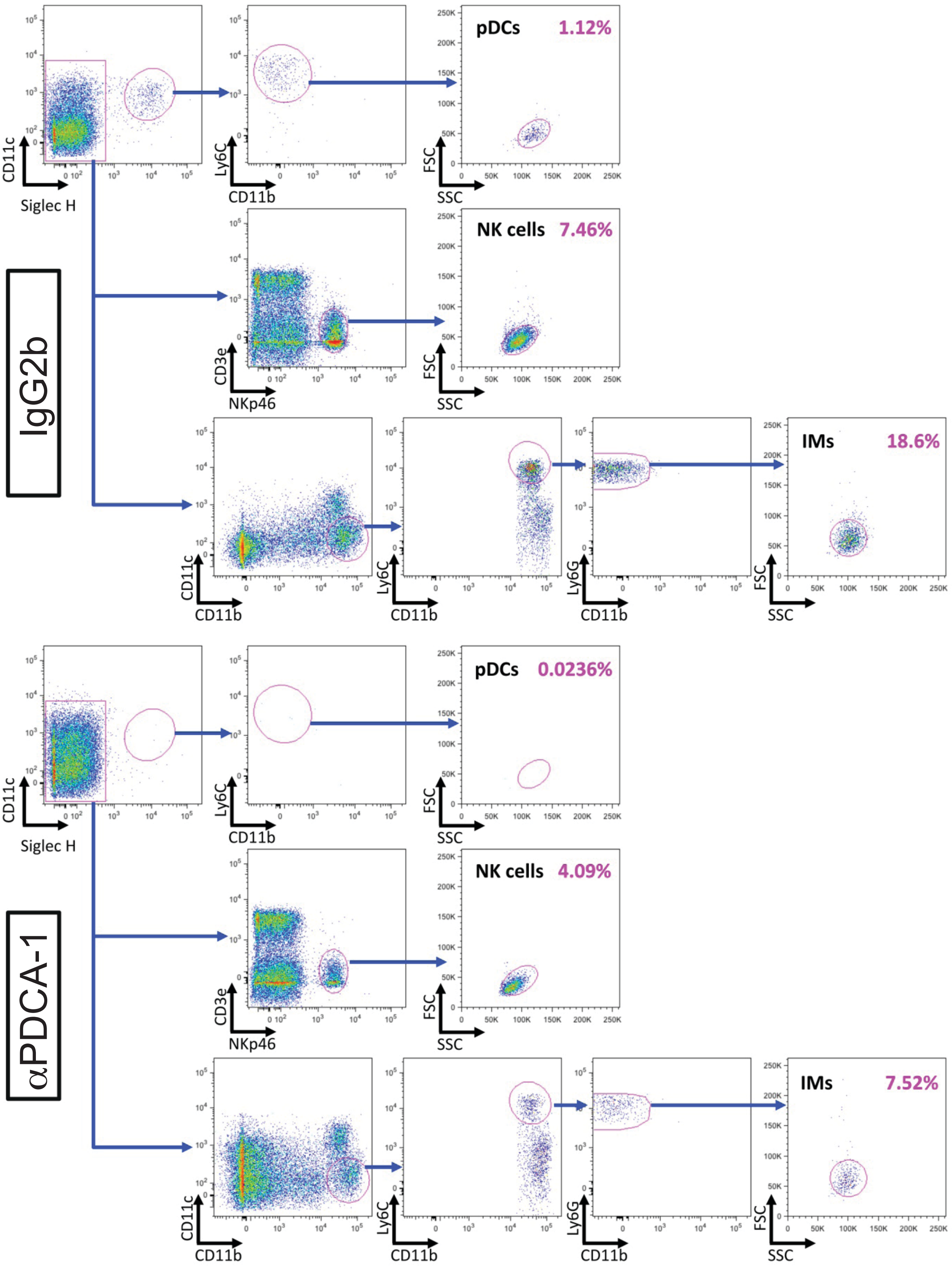

Supplementary Figure 6: αPDCA-1 mAbs effectively depletes pDCs and changes frequencies of cells present in the lung of infected 129S7 mice.
129S7 mice were treated with the pDC-depleting mAb αPDCA-1 (500µg/200µL i.p) or with isotype control (IgG2b) as indicated, and lung single cell suspensions were prepared. Gating strategies for flow cytometric quantification of pDCs (Siglec H+, CD11c+, Ly6C+, CD11b-, FSClo, SSClo), NK cells (NKp46+, CD3-, FSClo, SSClo) and inflammatory monocytes (Siglec H-, CD11b+, CD11c-, Ly6Chi, Ly6G- FSCint, SSCint) depicted using Flow Jo Version 9.5. Percentages indicate percent of live cells.

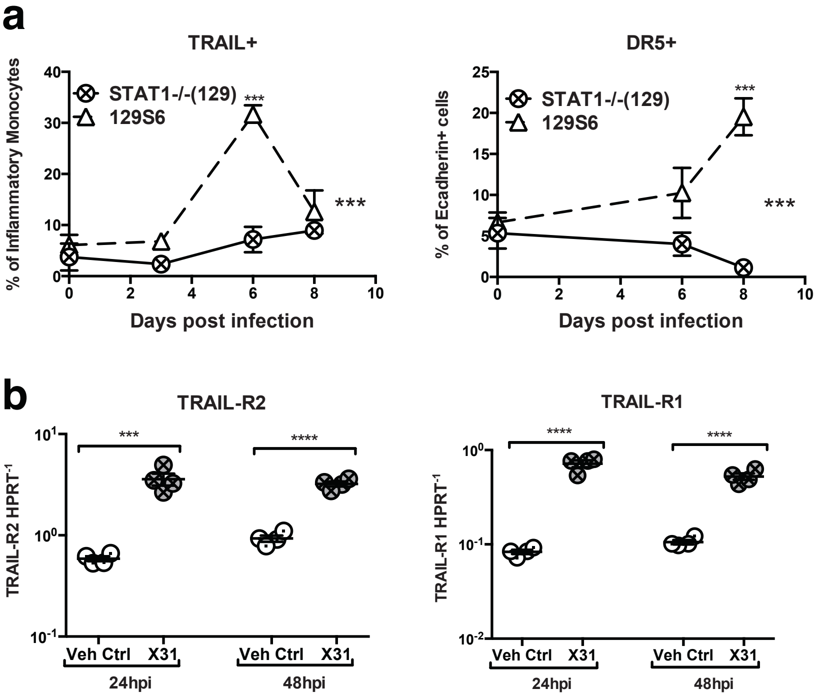

Supplementary Figure 7: DR5 and TRAIL expression during influenza infection in STAT1-/- mice and human epithelia.

(a) 129S6 and STAT1-/-(129) mice were infected intranasally with X31, lung single cell suspensions were prepared at indicated time points after infection, and flow cytometric quantification of inflammatory monocytes (Ly6Chi, CD11b+, FSCint, SSCint, + or – for TRAIL) and epithelial cells (CD45-, Ecadherin+, + or - for DR5) in the lung was performed. **(b)** A549 cells were infected with X31 (or vehicle control) and assessed for expression of TRAIL receptors 2 and 1 (DR5 and DR4, respectively) by qPCR at specified time points. Graphs show mean ± s.e.m and are representative of 2 pooled experiments where n=2-4. Significance (*** p<0.0001, ** p<0.001, * p<0.01) tested by 2-way ANOVA with Bonferroni post tests or students T test.

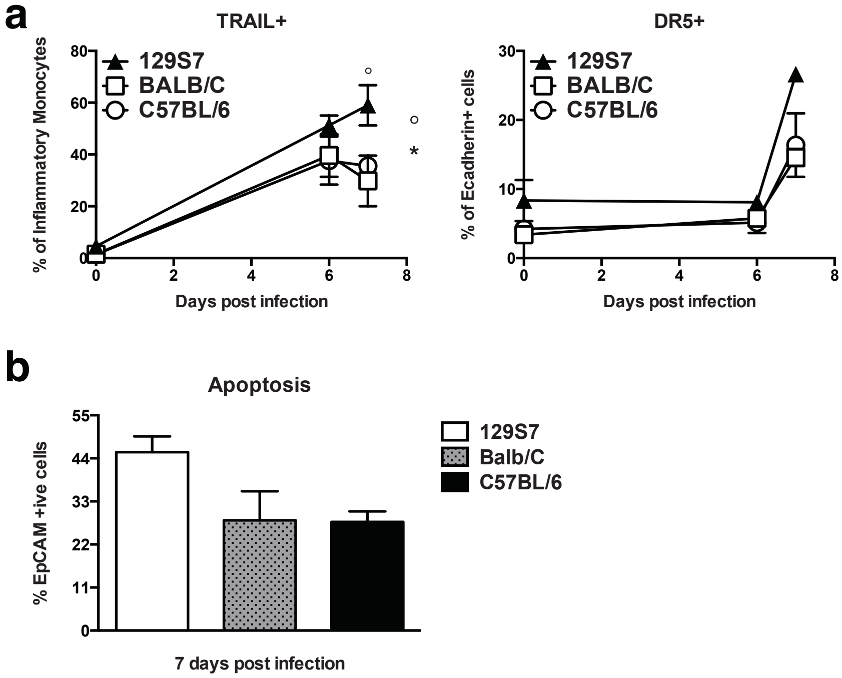

Supplementary Figure 8: Low type I IFN producing mouse strains: C57BL/6 and BALB/c express comparatively less TRAIL and DR5 during influenza infection.
129S7, C57BL/6 and BALC/c mice were infected intranasally with X31 and lung single cell suspensions were prepared at indicated time points after infection. **(a)** Flow cytometric quantification of TRAIL expression on inflammatory monocytes (Ly6Chi, CD11b+, FSCint, SSCint) and DR5 expression on epithelial cells (CD45-, Ecadherin+) in the lung was performed. **(b)** 7dpi epithelial cells were assessed for DR5 expression and apoptosis. Graphs show mean ± s.e.m and are representative of 2 independent experiments where n=3. Significance (*** p<0.0001, ** p<0.001, * p<0.01) tested by 2-way ANOVA with Bonferroni post tests.

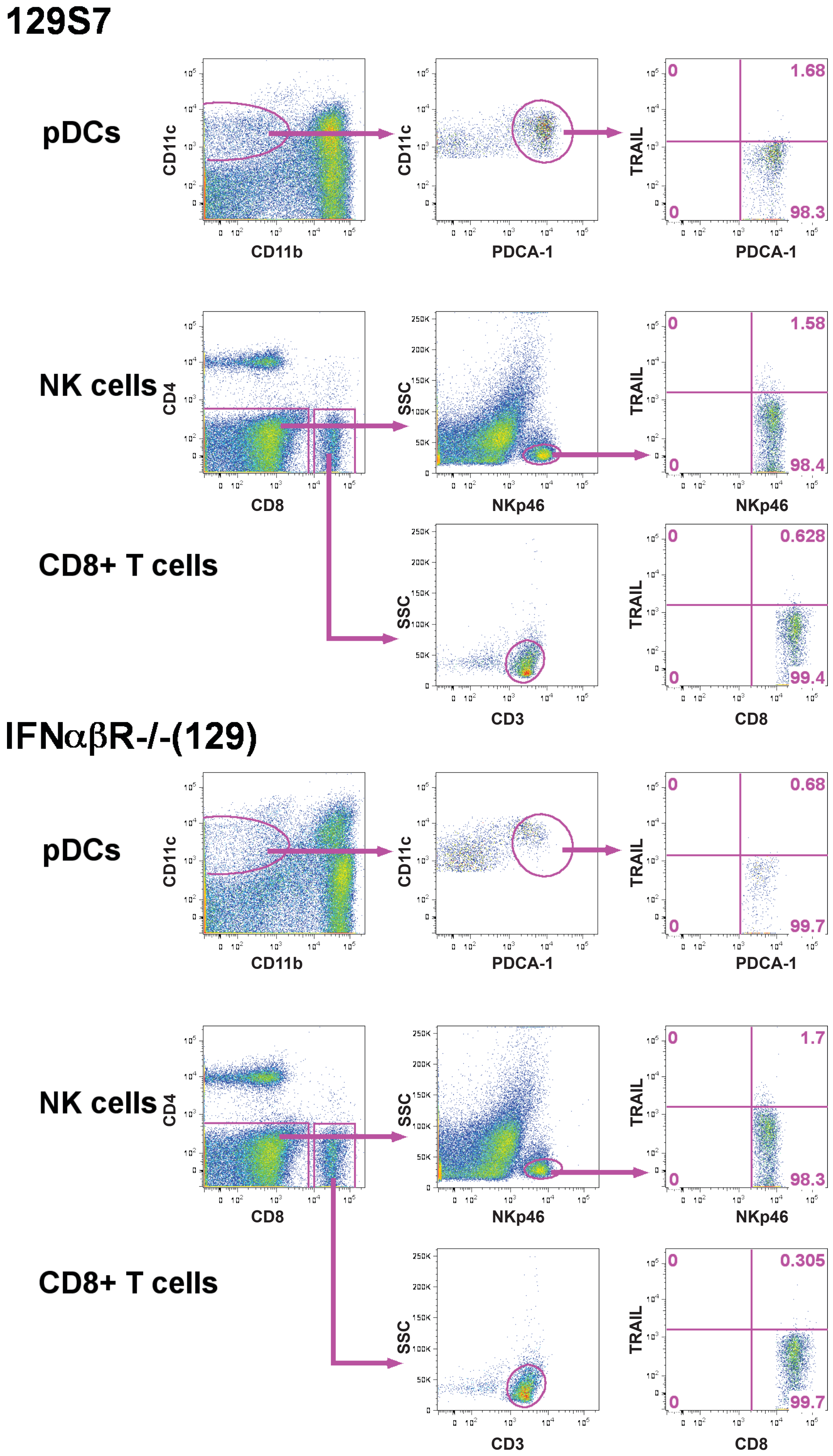

Supplementary Figure 9: pDCs, NK cells and CD8+ T cells do not significantly upregulate TRAIL during influenza infection.
129S7 and IFNαβR-/-(129) mice were infected with X31 and at 6dpi lung single cell suspensions were prepared. Gating strategies for flow cytometric quantification of TRAIL expression on pDCs (CD11b-, CD11c+, PDCA-1+, FSClo, SSClo), NK cells (NKp46+, CD4-, CD8-, FSClo, SSClo) and CD8+ T cells (CD8+, CD4-, CD3+, FSClo, SSClo) depicted using Flow Jo Version 9.5.

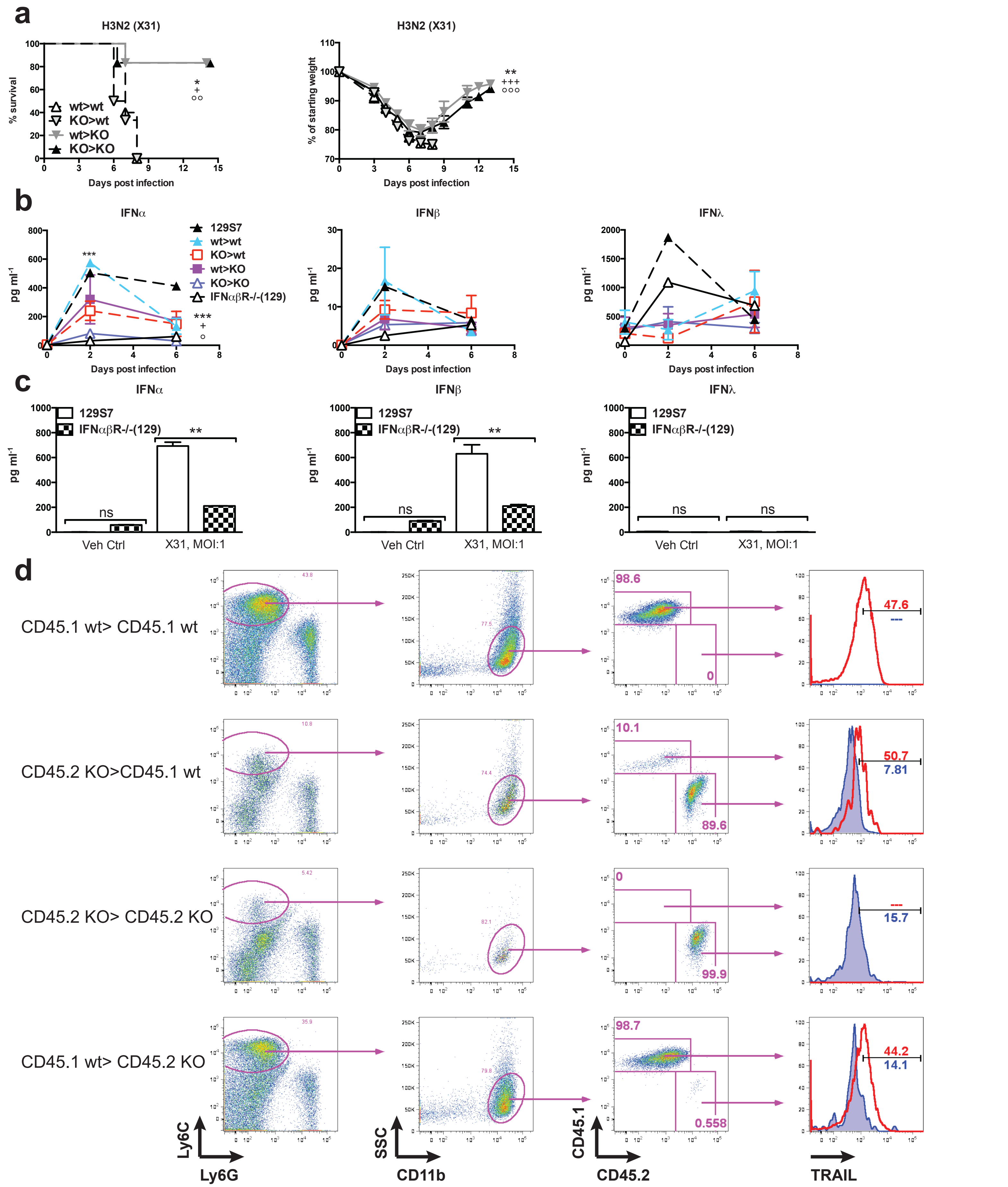

Supplementary Figure 10: Upregulation of TRAIL on inflammatory monocytes is dependant upon a type I IFN signal.

(a-b, d) BM chimeras were generated: wt>wt, KO>wt, wt>KO and KO>KO and with IFNaßR-/-(129) and 129S7 mice infected with X31. **(a)** Weight loss and survival was recorded, **(b)** IFN concentration in BAL was measured by ELISA. **(d)** At 6dpi lung single cell suspensions were prepared, and expression of TRAIL on inflammatory monocytes was assessed by flow cytometry. Gating strategy depicted using Flow Jo Version 9.5, red histograms indicate CD45.1+ wt 129 cells and blue indicate IFNαβR-/-(129) CD45.2+ cells. **(c)** Bone Marrow derived macrophages from 129S7 and IFNαβR-/-(129) mice were stimulated with X31, MOI of 1 for 24hrs, concentrations of IFNs in supernatants were then assessed by ELISA. Graphs show mean ±s.e.m and are representative of 2 independent experiments where **(a)** n=6 and n=2-3 for **(b-c)**. Significance (*** $p<0.0001$, ** $p<0.001$, * $p<0.01$) tested by (a) 2-way ANOVA with Bonferroni post tests or (b) student's T test where * denotes wt>wt:KO>KO, + for wt>wt:wt>KO and ○ represents for KO>KO: KO>wt.

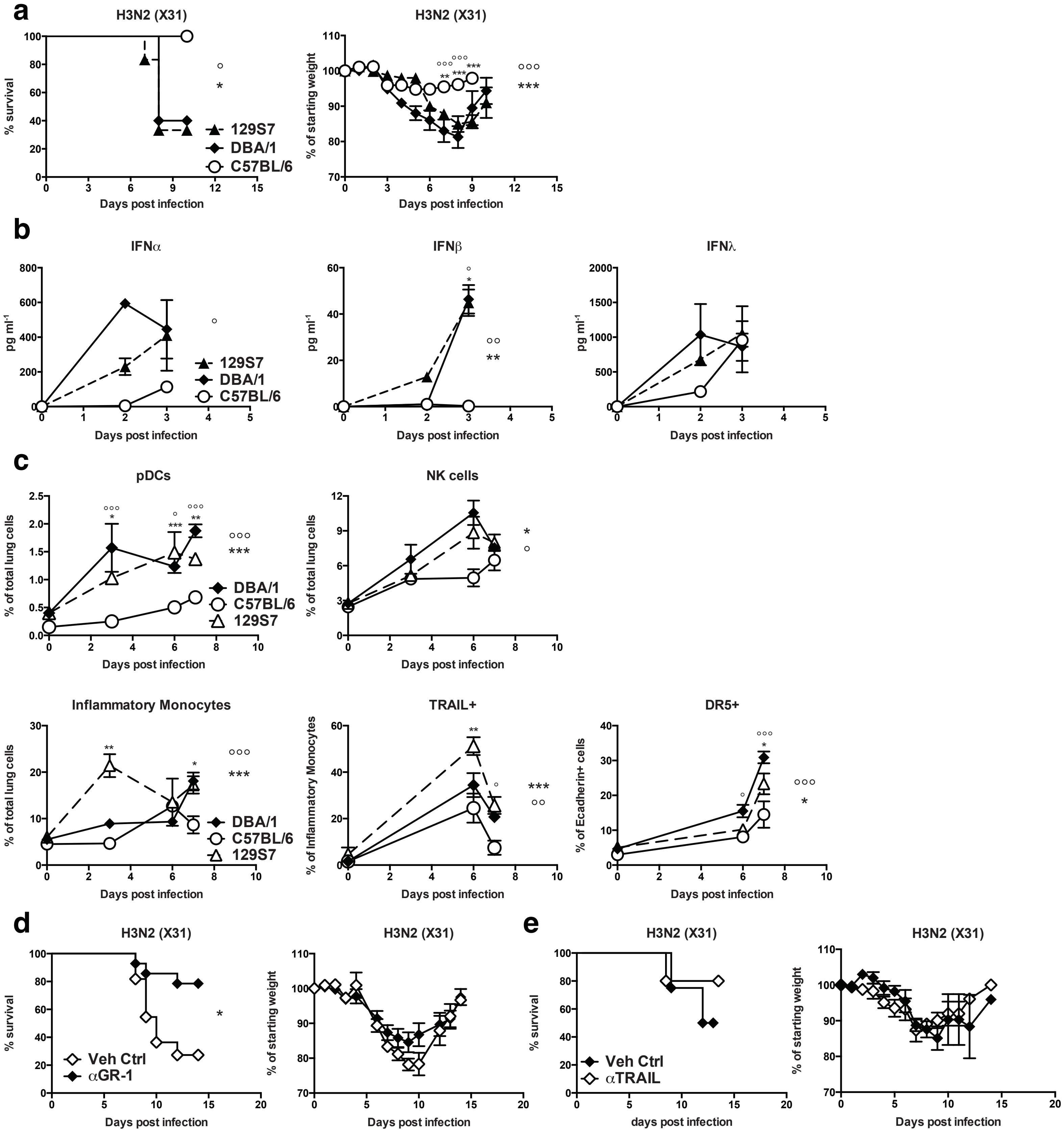

Supplementary Figure 11: DBA/1 strain susceptibility to influenza induced disease follows high type I IFN production, pDC and iMC recruitment and TRAIL/DR5 expression.
129S7, C57BL/6, and DBA/1 mice were infected i.n with X31. **(a)** Weight loss and mortality were recorded and **(b)** IFN concentrations BAL measured by ELISA. **(c)** At specified time points lung single cell suspensions were prepared, and recruitment of pDCs, NK cells and iMCs, expression of TRAIL on iMCs and DR5 on epithelial cells were assessed by flow cytometry. **(d-e)** DBA/1 mice were treated **(d)** αGr-1 or Veh ctrl or **(e)** αTRAIL or Veh ctrl (as indicated) and infected with X31, weight loss and mortality was recorded throughout infection. Graphs show mean ± s.e.m and are representative of 2 independent experiments where n=2-4 for cellular recruitment and ELISA, n=5-6 for weight loss and survival except for **(d)** where data is pooled from two experiments (n=15). B6:129S7 *, and B6:DBA/1: ○, where *** p<0.0001, ** p<0.001, * p<0.01 by 2-way ANOVA with Bonferroni post tests (weight loss and ELISA) or Log-rank (Mantel-Cox) Test (survival).
